# Bypass of complex co-directional replication-transcription collisions by replisome skipping

**DOI:** 10.1093/nar/gkab760

**Published:** 2021-09-01

**Authors:** Jan-Gert Brüning, Kenneth J Marians

**Affiliations:** Molecular Biology Program, Memorial Sloan Kettering Cancer Center, 1275 York Avenue, New York, NY 10065, USA; Molecular Biology Program, Memorial Sloan Kettering Cancer Center, 1275 York Avenue, New York, NY 10065, USA

## Abstract

Collisions between the replisome and RNA polymerases [RNAP(s)] are the main obstacle to DNA replication. These collisions can occur either head-on or co-directionally with respect to the direction of translocation of both complexes. Whereas head-on collisions require additional factors to be resolved, co-directional collisions are thought to be overcome by the replisome itself using the mRNA transcript as a primer. We show that mRNA takeover is utilized primarily after collisions with single RNAP complexes with short transcripts. Bypass of more complex transcription complexes requires the synthesis of a new primer downstream of the RNAP for the replisome to resume leading-strand synthesis. In both cases, bypass proceeds with displacement of the RNAP. Rep, Mfd, UvrD and RNase H can process the RNAP block and facilitate replisome bypass by promoting the formation of continuous leading strands. Bypass of co-directional RNAP(s) and/or R-loops is determined largely by the length of the obstacle that the replisome needs to traverse: R-loops are about equally as potent obstacles as RNAP arrays if they occupy the same length of the DNA template.

## INTRODUCTION

The accurate duplication of genomic material is a prerequisite for faithful cell division and the generation of viable progeny. However, during DNA replication, the replication machinery must overcome a range of obstacles that block replisome progression, such as DNA template damage or DNA–protein complexes (1–3). In bacteria, rapid DNA replication (at ∼900 nt/s) and slow RNA transcription (at ∼50 nt/s) are not separated temporally. Thus, collisions between the replisome and RNA polymerases [RNAP(s)] are unavoidable and are the main source of replication stalling *in vivo* (3,4). Even in eukaryotes, where replisomes and RNAPs translocate at similar rates, collisions still occur, given that transcription of some genes takes longer than an entire cell cycle (5).

Whereas head-on collisions between the replisome and RNAPs are thought to be most detrimental to genome stability, it was shown that co-directional (CO) replication-transcription collisions with a single RNAP were quickly overcome (6–9). Bypass involved the leading-strand DNA polymerase using the nascent mRNA transcript as a new primer (a mechanism named ‘mRNA takeover’), which resulted in the formation of a short gap in the nascent leading strand (6). However, replication-transcription collisions are likely more complex *in vivo*. RNAPs often stall during transcription and in some cases can ‘backtrack’ on the transcript resulting in the loss of the 3′-OH of the nascent transcript from the active site and making these backtracked RNAPs more potent sources of genome instability (10–13). Furthermore, RNAPs often stall at sites of DNA damage *in vivo* that the replisome cannot bypass easily (14–18). In the case of CO replication-transcription collisions, any DNA lesion blocking a RNAP is also encountered by the leading-strand polymerase, making resolution via mRNA takeover impossible. Thus, it is unlikely that all CO collisions between the replisome and RNAPs can be resolved simply by mRNA takeover, suggesting that other bypass mechanisms must exist to continue DNA replication past such obstacles to avoid genome instability.

Indeed, bypass of replication obstacles can be promoted by accessory helicases that have been found in multiple organisms (2,19,20). These helicases promote the removal of replication barriers, such as protein–DNA complexes like RNAPs and reduce the formation of single-stranded (ss) DNA gaps and replisome collapse (19,21,22). Additionally, transcription-coupled repair factors monitor the genome for stalled RNAPs to remove them from the DNA template before they are encountered by an approaching replisome (23–25). This is particularly advantageous to prevent genome instability, as the formation of ssDNA gaps as a consequence of replisome bypass of CO RNAP complexes has been shown to induce double-stranded (ds) DNA breaks if these gaps are not filled by the time a second replisome approaches (13).

The introduction of negative supercoils behind translocating RNAPs can promote the rehybridization of the mRNA transcript to the template strand, resulting in the formation of R-loops (26,27). R-loops are potent sources of genome instability, causing replication fork stalling and DNA breaks (28–30). Although we have observed the formation of ssDNA gaps as a result of replisome collisions with short RNAP-free R-loops previously *in vitro* (31), studies *in vivo* have attributed little or no significant effect of CO R-loops on genome stability (32,33). However, it is not known if RNAP-associated CO R-loops have any additional effect on replisome bypass compared to an RNAP that is not associated with an R-loop. It is possible that resulting ssDNA gaps are quickly repaired *in vivo*, or that other mechanisms within cells efficiently deal with problems arising because of R-loops via the activity of topoisomerases or active removal of R-loops by RNase H or helicases (8,34–37).

Using a previously established *in vitro* replication system (31), we have defined the mechanisms by which the *E. coli* replisome bypasses CO replication-transcription collisions of varying complexity. We show that mRNA takeover is utilized primarily for collisions with a single RNAP with a short extendable transcript. In the case of more complex CO replication-transcription collisions, replisomes skip over the obstacle by resynthesizing a new primer downstream on the leading-strand template and continuing leading-strand synthesis, leaving a ssDNA gap behind, with mRNA takeover playing a minor role. In both cases, the RNAP is displaced from the template. Other factors such as the accessory helicases Rep and UvrD, the translocase Mfd, and RNase H can also promote the formation of a continuous leading strand, indicating that multiple mechanisms exist to avoid the formation of longer ssDNA gaps *in vivo*. Furthermore, we show that R-loops formed behind RNAPs increase the severity of the CO collisions. R-loops alone are roughly equally potent replication obstacles as an RNAP array that occupies a similar length of the DNA template, suggesting that a major determinant of replisome stalling is the overall distance the obstacle occupies on the template, whether it is an R-loop or RNAP(s).

## MATERIALS AND METHODS

### Purification of replication templates

**Figure 1.**
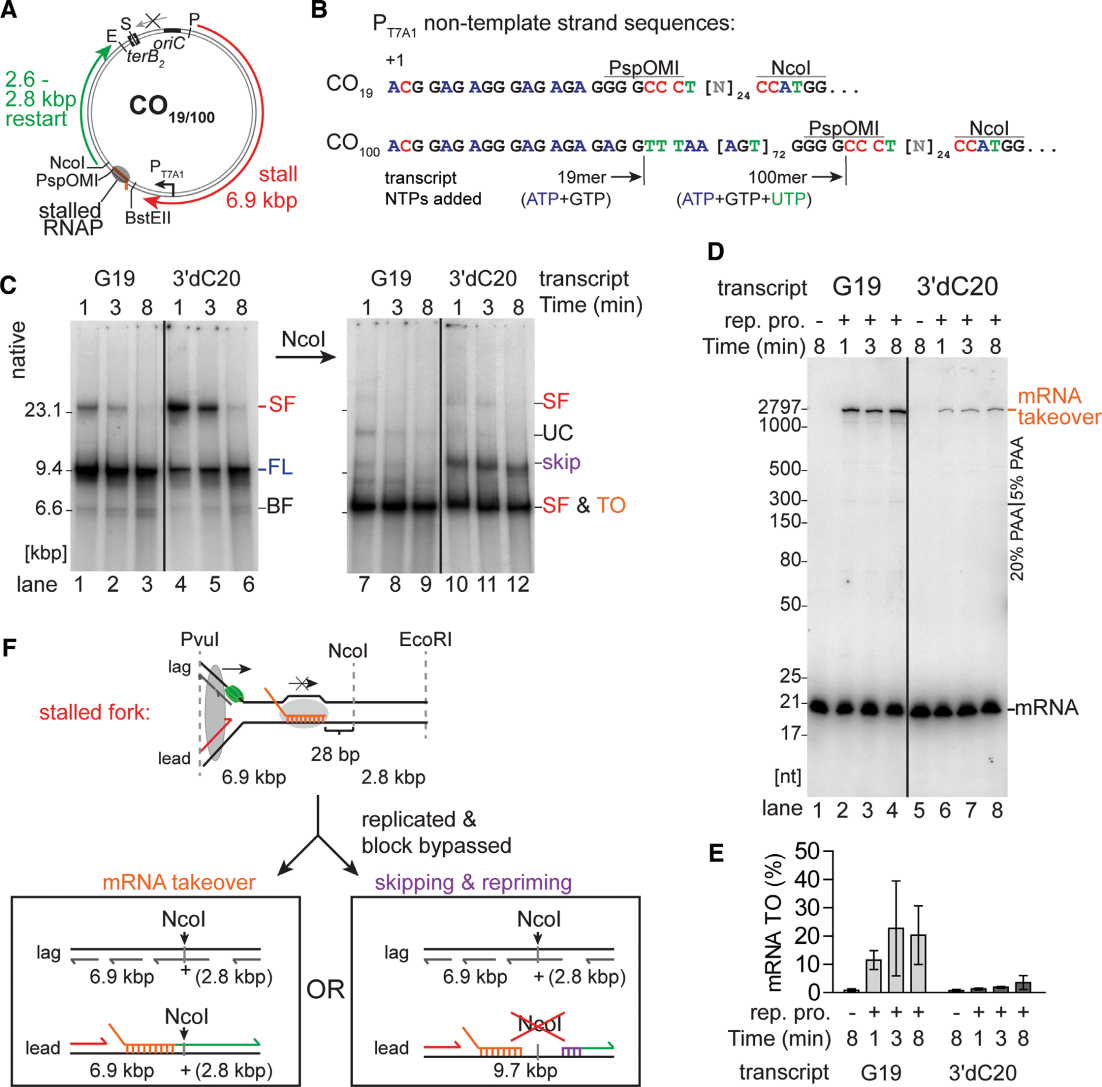
Replisome skipping resolves CO collisions if mRNA takeover is blocked. (**A**) Schematic representation of the replication template with relevant features, restriction sites and leading-strand product lengths. (**B**) Sequence of the T7 A1 promoter non-template sequences for both replication templates with relevant transcript lengths and restriction sites. (**C**) Native gel electrophoretic analysis of the products of a replication time course of CO collisions with a single RNAP with a 19mer mRNA transcript either with a 3′-OH group (G19) or a 20mer transcript without a 3′-OH group (3′dC20) on the CO_19_ template (*n* = 3). Replication efficiency averaged 11 ± 6% (1 min)—13 ± 7% (8 min) for G19 and 10 ± 5% (1 min)—15 ± 7% (8 min) for 3′dC20. Lanes 1-6, reaction products digested with EcoRI and PvuI only; lanes 7–12, replication products were additionally digested with NcoI. (**D**) Gel-filtered [α-^32^P]GMP-labeled 19mer-RNAP CO_19_ templates with either a free 3′-OH end (G19; lanes 1–4) or terminated with 3′-dCTP (3′dC20; lanes 5–8) were used in standard replication reactions and the products analyzed by electrophoresis through a composite 5%/20% 7 M urea–polyacrylamide gel. (**E**) Fraction of the labeled RNA extended by mRNA takeover on the gel shown in panel D (*n* = 3, mean ± standard deviation). (**F**) Schematic representation of possible replication outcomes with respect to NcoI digestion. E, S and P, EcoRI, ScaI and PvuI restriction sites, respectively; rep. pro., replication proteins; SF, stalled fork; FL, full-length product; BF, broken fork; UC, uncoupled product (14); skip, NcoI-resistant material resulting from replisome skipping; TO, mRNA takeover product; dark grey oval, replisome; light grey ovals, RNAP.

Replication templates CO_19_ and CO_100_ (Figure 1A) were purified from cells by alkaline lysis, ethidium bromide-CsCl (1 g/ml) density gradient centrifugation and 15–35% sucrose gradient centrifugation as described previously (31).

### Replication and transcription proteins

Replication proteins were purified as described previously: RNA polymerase core (38); σ^70^ (39); DnaA and HU (40); DnaB, DnaC, and DnaG (41); DnaN (β clamp) (42); Pol III* (43); SSB (44); Tus (45); DNA Gyrase (46); UvrD, gift of T. Lohman. RNase H was purchased from NEB. RNase A (Roche, 100 mg/ml in 10 mM Tris–HCl, pH 8.0, 1 mM EDTA) was heated for 5 min at 95°C and stored at 4°C.

Mfd was expressed from pET11a-*mfd* (full length *mfd* open reading frame inserted into the NdeI and BamHI sites of pET11a) in BL21(DE3). Cells were grown in LB medium supplemented with 100 μg/ml ampicillin to an O.D._600_ of 0.8 before induction with 1 mM IPTG at 37°C for 3 h. Harvested cells were resuspended in 50 mM Tris–HCl (pH 7.5)-10% sucrose and lysed in 50 mM Tris–HCl pH 8.8, 250 mM KCl, 20 mM EDTA, 2 mM DTT, 20 mM spermidine and 0.4 mg/ml lysozyme on ice for 20 min followed by the addition of Brij-58 (0.1% final) for 30 min before centrifugation at 100 000 × g for 1 h. The supernatant was diluted with buffer A (50 mM Tris–HCl, pH 8, 1 mM EDTA, 1 mM DTT, 10% glycerol, 1 mM PMSF) to match the conductivity of buffer A containing 0.025 M NaCl and purified with a linear gradient of 0.025 M to 0.5 M NaCl in buffer A on a Q-Sepharose FF column (15 mg protein/ml resin). Fractions containing Mfd, eluting at about 0.1 M NaCl, were pooled and diluted with buffer A containing 2.5 M NaCl to match the conductivity of buffer A containing 1.5 M NaCl and further purified with a linear gradient of 1.5 M to 0.05 M NaCl in buffer A on a butyl-Sepharose FF column (5 mg protein/ml resin). Fractions containing Mfd, eluting at about 1 M NaCl, were pooled and concentrated by (NH_4_)_2_SO_4_ precipitation (50% saturation). The precipitate was resuspended in 2 ml buffer A containing 0.5 M NaCl and chromatographed on a Superdex S200 16/600 column equilibrated and developed in buffer A containing 0.5 M NaCl. Fractions containing Mfd were pooled and applied to a hydroxylapatite-CF11 cellulose (60:17) column (5 mg protein/ml resin). Mfd was eluted with a linear gradient of 0.1 M to 0.8 M (NH_4_)_2_SO_4_ in buffer A containing 0.1 M NaCl. Fractions containing Mfd, eluting at about 0.3 M (NH_4_)_2_SO_4_, were pooled (Fr. V), dialyzed against 50 mM Tris–HCl (pH 8.0), 1 mM EDTA, 1 mM DTT, 40% glycerol, and 0.2 M NaCl, and stored in aliquots at −80°C.

### Transcription and replication reactions

Transcription reactions were performed and R-loop templates prepared as described previously (31). Briefly, to form a single RNAP with a 19mer transcript on template CO_19_, transcription reaction mixtures contained only 250 μM ApC (TriLink) and 0.5 μM ATP and GTP as ribonucleotides. For incorporation of the chain terminator, 250 μM 3′-dCTP (TriLink) was added 1 min after transcription was initiated by the addition of RNAP holoenzyme (200 nM RNAP core + 1 μM σ^70^). The single RNAP with a 100mer was formed on template CO_100_, initially with only ApC, ATP and GTP to form a 19mer. After 8 min of incubation, the reaction mixtures were spin-dialyzed to remove free RNAP and the template-associated 19mer transcripts were extended into 100mers by adding ATP, GTP and UTP to a final concentration of 10 μM (and 3′-dCTP to 250 μM if the 100mer was to be chain terminated). The RNAP array was formed on template CO_100_ with 250 μM ApC, and 10 μM of ATP, GTP, and UTP present from the start. For visualization of mRNA transcripts, [α-^32^P]GTP was added to 0.05 μM before the start of the transcription reactions. RNAP-free templates were inactivated for DNA replication initiation by PspOMI digestion (NEB, 0.5 U/μl) for 10 min. This enzyme cannot cut the template when RNAPs are bound at its recognition sequence (Figure 1B). The RNAP-DNA complexes were then isolated by gel filtration as described previously (14).

Replication reactions were performed as described previously (31). The reactions were ScaI-HF digested (NEB, 0.33 U/μl) 40 s after replication was initiated to generate pseudo-synchronous replication fork progression, unless stated otherwise. Aliquots (8 μl) were taken at the indicated times and incubated for 10 min with 0.2 U/μl EcoRI-HF (NEB), 0.2 U/μl PvuI-HF (NEB), 2 mM AMP-PNP (Roche), and 133 μM ddNTPs (GE Healthcare) (‘stop buffer’) before further analysis by gel electrophoresis.

#### Calculation of replication efficiency and distribution of replication products

**Figure 2.**
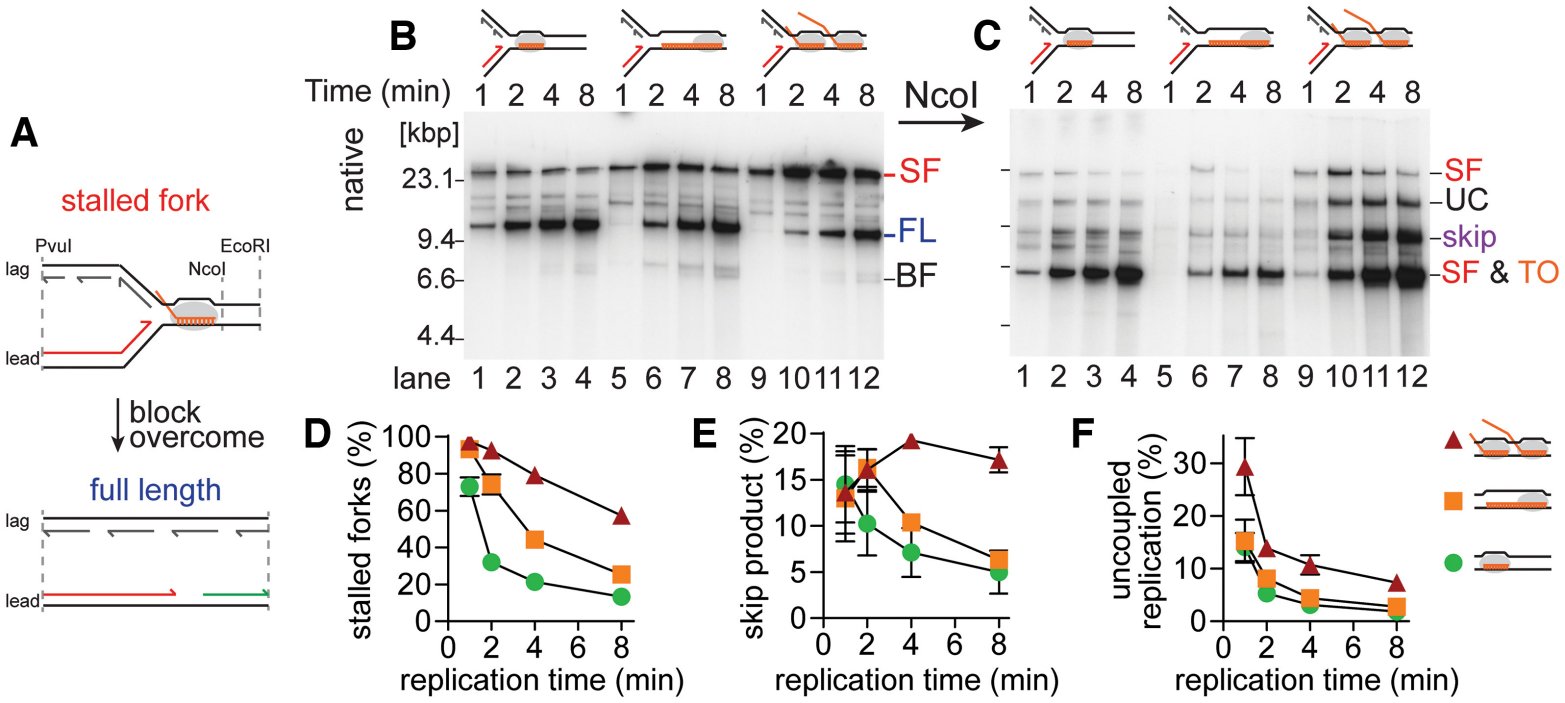
Replisome skipping predominates as CO RNAP collision complexity increases. (**A**) Cartoon describing different possible replication outcomes. Native agarose gel of products in replication time courses of CO replication collisions with the indicated RNAP template complexes formed on template CO100 either (**B**) without or (**C**) with additional NcoI digestion, as described in the legend to Figure 1F. Quantification of (**D**) stalled forks from the gel shown in panel B and (**E**) replisome skip products, and (**F**) uncoupled products from the gel in panel C (*n* = 5, mean ± standard deviation). Lanes 1–4, 19mer RNAP template, •; lanes 5–8, 100mer RNAP template, ▪; lanes 9–12, RNAP array template, ▴. Replication efficiency and fraction of DNA products as full length were calculated as described in the methods section. Note that the plot shown in (D) is the same as we published previously (31), whereas the gel shown in (B) is different. SF, stalled fork; FL, full-length product; BF, broken fork; UC, uncoupled product (14); skip, NcoI-resistant material resulting from replisome skipping; TO, mRNA takeover product; grey ovals, RNAP.

Replication efficiency is defined as the observed incorporation of [α-^32^P]dATP precursor into acid-insoluble product (as determined by precipitation of an aliquot of the reaction mixture after termination of the replication reaction with trichloroacetic acid) divided by the maximum possible incorporation (as determined by the nucleotide sequence of the template DNA). Thus, a replication efficiency of 20% indicates that 20% of the total number of templates in the reaction were replicated. Note that replication efficiency and the fraction that any particular replication product represents of the total of replication products are two distinct values. The latter value is determined by phosphorimager analysis of the distribution of all replication products in the gel, corrected for the relative lengths of the radioactive DNA products. Also note that full-length products on native and denaturing agarose gels do not necessarily correlate. On native gels, the bands represent double-stranded DNA molecules of different shapes (e.g. Y-shaped or linear, Figure 2A) where the template strands are hybridized to nascent un-ligated Okazaki fragments and leading strands that may be synthesized discontinuously if a lesion is encountered. On the denaturing gels, the nascent DNA strands are separated from the template strands and only the radioactively labeled nascent leading strands and lagging-strand Okazaki fragments are visualized.

#### Assaying replisome skipping

When indicated, NcoI-HF (NEB) was added to the stop buffer to 0.2 U/μl (Figures 1C and 2C).

#### Promotion of collision bypass by proteins

To promote bypass of RNAP-replication fork collisions, Rep (100 nM final), UvrD (100 nM), Mfd (500 nM), RNase H (0.1 U/μl) or RNase A (20 μM) were added to pooled RNAP-DNA complexes either for 10 min before the addition of replication proteins (Figures 5, 6 and Supplementary Figure S6) or added with the replication proteins (Supplementary Figures S4 and S6).

#### Visualizing replication intermediates

After incubation of the replication reaction mixtures for the indicated times, the reactions were terminated by the addition of EDTA to 30 mM. DNA products were not digested with any restriction enzyme and were analyzed either by native gel electrophoresis or by denaturing PAGE as described previously (31).

#### Mapping of nascent leading strands

To generate a short nascent leading-strand fragment, pooled RNAP-DNA complexes were either not treated with RNase, or treated with either 0.1 U/μl RNase H or 20 μM RNase A for 10 min before standard replication reactions were performed. The DNA products were additionally digested in stop buffer containing BstEII-HF (NEB, 0.2 U/μl). DNA products were then phenol-chloroform-isoamyl alcohol extracted, ethanol precipitated, and analyzed via electrophoresis through a 10% 7M urea polyacrylamide gel.

#### Estimating RNAP dissociation by western blot analysis

**Figure 3.**
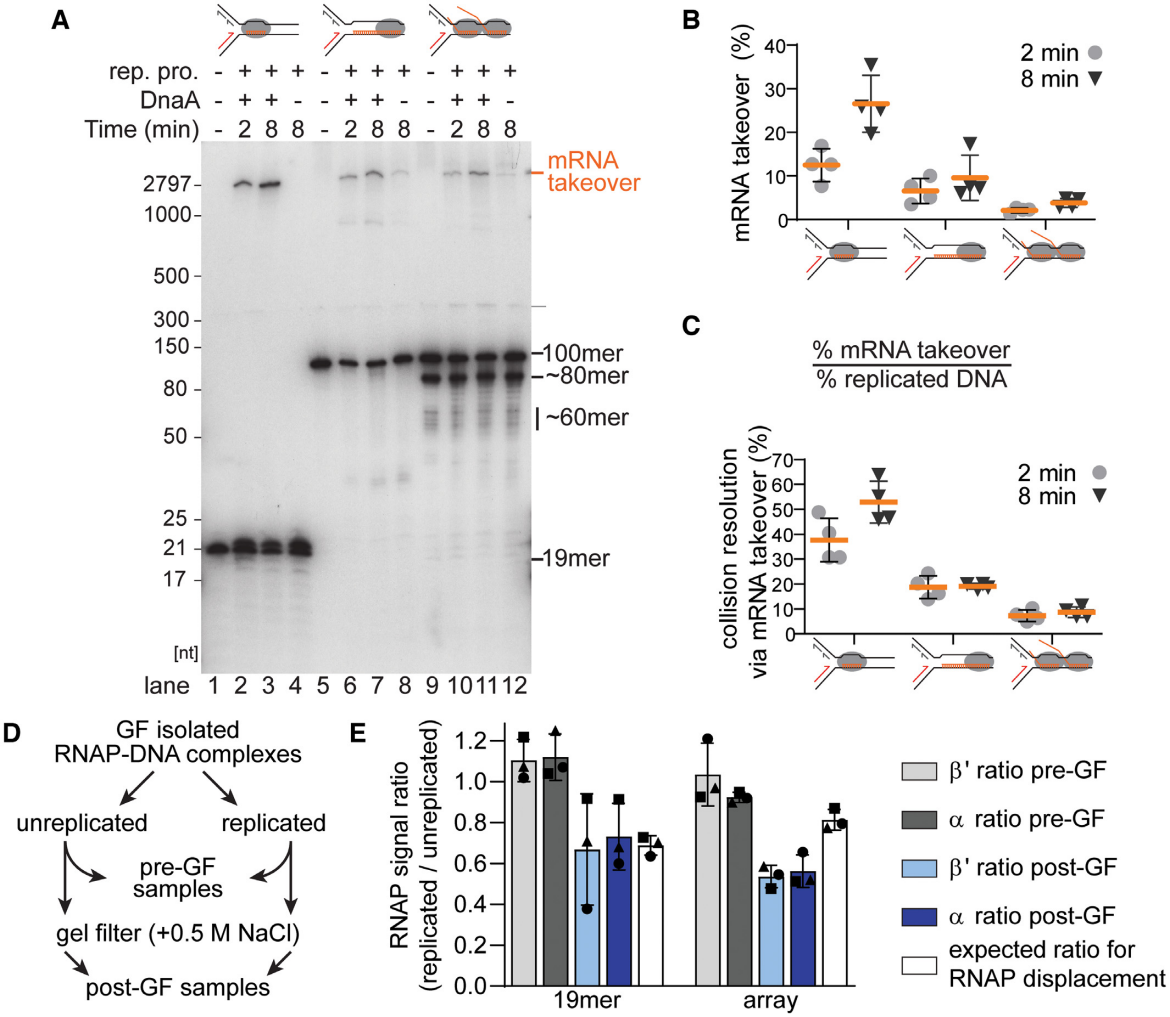
RNAP bypass by mRNA takeover is reduced as the complexity of CO replication-transcription collisions increases. (**A**) Replication reaction mixtures on template CO100 containing [α-^32^P]GMP-labeled 19mer-RNAP (lanes 1–4), 100mer RNAP (5–8), and RNAP arrays (9–12) were incubated for the indicated times with or without *E. coli* replication proteins except DnaA (rep. pro.) and with or without DnaA. The products were analyzed by electrophoresis through a composite 5%/20% 7 M urea polyacrylamide gel. (**B**) Fraction of the labeled RNA extended by mRNA takeover on the gel shown in panel A. (**C**) mRNA takeover (%) relative to the extent of replication measured by [α-^32^P]dAMP incorporation in independent replication reactions using the same RNAP template preparations as in panel A. Replication efficiency averaged 34 ± 12% (1 min)—52 ± 15% (8 min) for the 19mer; 36 ± 21% (1 min)—51 ± 25% (8 min) for the 100mer; and 30 ± 8% (1 min)—45 ± 12% (8 min) for the RNAP array (*n* = 4, mean ± standard deviation). (**D**) Assay protocol to estimate RNAP displacement by active DNA replication. (**E**) Ratios of β′ and α RNAP subunit signal intensities from western blots (replicated/unreplicated) in reactions before and after gel filtration in high salt compared to the RNAP subunit ratio predicted for active RNAP dissociation by replisome bypass (*n* = 3, mean ± standard deviation). A more detailed analysis of one of the experimental repeats is shown in Supplementary Figure S3. Gray ovals, RNAP.

Standard *in vitro* transcription reactions were performed, PspOMI digested, and gel filtered through a 3 mm × 195 mm Sepharose 4B column equilibrated and developed in GF buffer (60 mM HEPES pH 8, 90 mM potassium glutamate, 12 mM DTT, 12 mM Mg(OAc)_2_, 0.015% IGEPAL CA-630, and 0.5 μM ATP and GTP). The pool (110 μl) of RNAP-bound templates was divided in two and one-half incubated without replication proteins and the other half with replication proteins in a final reaction volume of 62 μl. In the case of RNase-treated samples (Figure 6E), RNase H (0.1 U/μl) and RNase A (20 μM) were added for 10 min prior to the addition of the replication proteins. Reactions were stopped after 8 min by the addition of 0.2 U/μl EcoRI-HF, 0.2 U/μl PvuI-HF, 0.2 U/μl ScaI-HF, 2 mM AMP-PNP, and 133 μM ddNTPs in a final volume of 75 μl. After further incubation for 10 min, the reactions were terminated by the addition of EDTA to 30 mM. Samples were taken to determine ‘pre GF’ RNAP subunit ratios (Figures 3D, 6F, and see below), to measure replication template utilization as described above, and for native agarose (Supplementary Figure S3A) and SDS polyacylamide gel electrophoresis. Native agarose gels were dried and the extent of full-length product formation determined as described above. The remainder of the reaction mixtures (∼55 μl) were each chromatographed through a 3 mm × 195 mm Sepharose 4B column equilibrated and developed in GF buffer containing 0.5 M NaCl (to prevent re-binding of dissociated RNAP to the template (47)) and 10 μM AMP-PNP (to inhibit ATP-dependent DNA replication proteins). Excluded fractions containing the DNA and RNAP-template complexes were pooled to recover most of the DNA (Supplementary Figure S3B) and together with the unfiltered ‘pre GF’ samples analyzed by 4–20% SDS-PAGE. The proteins on the SDS-polyacrylamide gels were transferred electrophoretically to a nitrocellulose membrane overnight. The membrane was cut below the 75 kDa marker band and the top half was blotted with antibodies to the β′ subunit of RNAP (BioLegend, 1:1000 dilution) and the bottom half with antibodies to the α subunit of RNAP (BioLegend, 1:500 dilution). RNAP subunits were visualized by blotting with Goat Anti-Mouse IgG (H + L)-HRP Conjugate (BioRad, 1:10 000 dilution), activated with ECL Western Blotting reagent (GE Healthcare), and imaged on a BioRad ChemiDoc XRS+ (Figure 6E and Supplementary Figure S3C).

To estimate RNAP displacement by active DNA replication (Figures 3E and 6F), the ratio of RNAP subunits was calculated as the fraction of the subunit signal in the replicated sample divided by that in the unreplicated sample. For the ‘pre-GF’ samples, this value is expected to be about 1, as the RNAP–DNA input was from the same pool. The subunit ratio for pooled samples after gel filtration in the presence of 0.5 M NaCl was corrected for DNA recovery from the column (Supplementary Figure S3B) and dilution of the samples. The theoretical RNAP dissociation resulting from replisome bypass was calculated by multiplying the replication efficiency by the fraction of the full-length signal DNA product on the native agarose gel and subtracting this value from 1 (Supplementary Figure S3A). To measure possible enhancement of RNAP dissociation because of RNase treatment (Figure 6G), the ratio of RNAP subunit signals in RNase treated versus untreated samples is plotted. A ratio of 1 indicates retention of RNAPs on the DNA after RNase treatment.

### Gel electrophoresis

Native agarose, denaturing agarose and denaturing polyacrylamide gel electrophoresis was performed as described in Brüning and Marians (31). For 2D gel analysis, two aliquots of a replication reaction were run in parallel on a native gel at 20–25 V for 380 V h. The next day, one lane was dried and imaged, while the other was excised and incubated in denaturing gel electrophoresis buffer with shaking for 5 h. The gel slab was turned by 90° and inserted into a wide well on a denaturing gel. Gels were electrophoresed at 20–25 V for a total of 380 V h and fixed.

### Gel imaging and presentation

Ethidium bromide-stained gels and western blots were imaged with a BioRad ChemiDoc XRS + system.

Gels containing radioactive samples were dried onto chromatography paper (GE Healthcare, 3030-861) and imaged with a Typhoon FLA 7000 phosphorimager (GE Healthcare) for quantitative analyses. Dried gels were also exposed to Amersham Hyperfilm MP (GE Healthcare, 28906843) and scanned for data presentation.

### Quantification and statistical analysis

Phosphorimages of dried radioactive gels were quantified using Image Gauge v. 4.0 (Fujifilm). DNA intensities from EtBr stained gels and the intensity of RNAP subunits was quantified via ImageLab v. 5.2 (BioRad). The number of replicates (*n*) for experiments is indicated in the Figure legends. Data are plotted as means ± standard deviations.

## RESULTS

### Replisome bypass of CO RNAPs switches from mRNA takeover to replisome skipping as the complexity of the block increases

We have recently developed an *in vitro* replication system that allows us to monitor replication collisions with either a single or multiple RNAP complexes with associated mRNA transcripts of up to 100 nt in length (31). RNAP transcribing from the bacteriophage T7 A1 promoter in the presence of the initiating dinucleotide ApC and ATP and GTP results in the formation of a 19mer transcript. On template CO_100_, this 19mer RNAP–template complex could be spin-dialyzed to remove free RNAP and the 19mer could then be extended to an 100mer by adding back ATP, GTP and UTP. Alternatively, adding ATP, GTP and UTP from the start of the transcription reaction resulted in the formation of an array of up to three RNAP complexes with a maximum transcript length of 100 nucleotides on template CO_100_ (Figure 1A and B) (31). RNAP-free templates were digested with PspOMI, whereas PspOMI digestion was prevented on templates where the RNAP blocked access to the restriction site (Figure 1B). The RNAP–DNA complexes were isolated by gel filtration and DNA replication was initiated from *oriC* in the presence of DNA gyrase and *E. coli* replication proteins. The clockwise moving replication fork encounters the stalled RNAP complexes in a CO orientation after about 6.9 kb, whereas the counter-clockwise moving fork is blocked by Tus bound to *terB* sites (Figure 1A). Using these different RNAP–DNA template complexes, we investigated the mechanisms by which CO replication-transcription collisions were resolved.

A previous study demonstrated that replisome bypass of a CO-oriented 19mer-RNAP complex occurred by the leading-strand DNA polymerase taking over the short mRNA transcript and using it as a primer (‘mRNA takeover’) (6). Leading-strand synthesis continued, leaving behind a ssDNA gap. However, these reactions lacked the primase, DnaG. Our previous work established that DnaG could synthesize a new primer downstream of a DNA lesion on the leading-strand template, thereby allowing the DNA polymerase to skip over the lesion, leave a ssDNA gap behind, and continue DNA replication downstream (‘replisome skipping’) (14,48). In either case, the nascent leading strand is discontinuous. We have established that replisome skipping of a template lesion occurs in the presence of ATP and GTP as the only NTPs in the reaction mixtures (required to maintain RNAP stalling) (31), allowing us to test whether replisome skipping could also effect bypass of RNAP-transcript complexes in our replication system.

mRNA takeover by the replisome is dependent on the presence of a 3′-OH group (6). To suppress mRNA takeover of the 19mer RNAP transcript (G19), we incorporated the chain terminator 3′-dCTP into [α-^32^P]GMP-labeled 19mer transcripts on template CO_19_ (Supplementary Figure S1A, 3′dC20). When the [α-^32^P]dAMP-labeled DNA products of the replication reaction were examined by native agarose gel electrophoresis, both DNA-RNAP templates showed two major DNA products: a slow-moving band corresponding to stalled forks (SF) and a faster-moving band representing full-length, linear product (FL) (Figure 1C). At early time points with the G19-RNAP template, replication products were already mostly full length, indicating that the stalled forks were resolved very quickly. In contrast, stalled forks persisted for some time with the 3′dC20-RNAP template but were eventually chased into full-length material (Figure 1C, compare lanes 4–6 with lanes 1–3). In replication reactions where only the mRNA was radioactively labeled with [α-^32^P]GMP, these RNAP-bound templates showed extension of the G19mer to a ∼2.8 knt fragment (Figure 1D and E), dependent on DNA replication initiation from *oriC* (Supplementary Figure S1B). Thus, the 2.8 knt product was formed via replication-dependent mRNA takeover. In contrast, mRNA takeover was reduced significantly with the chain-terminated 3′dC20mer (Figure 1D and E). The small amount of residual takeover in the presence of 3′-dCTP presumably arises because of either incomplete incorporation of the chain terminator or a combination of RNAP backtracking followed by exonuclease trimming by Pol III*. These data suggested that resolution of the CO collisions with a chain-terminated transcript occurred via a mechanism other than mRNA takeover. We had previously eliminated one possible alternative mechanism, direct displacement of the RNAP and R-loop by the replisome (31), as considered further in the Discussion.

We suspected that this alternative mechanism of RNAP bypass was replisome skipping. To test this premise, replication products were digested with NcoI. The recognition sequence for this enzyme is 28 bp downstream of the stalled RNAP. mRNA takeover would generate nascent leading- and lagging-strand sister molecules that are double-stranded at this location. NcoI digestion would therefore convert the 9.6 kb full-length product to 6.9 kb and 2.8 kb products. However, should collision resolution involve replisome skipping downstream of the RNAP block (and also the NcoI site), NcoI digestion across the ssDNA gap would not occur (Figure 1F). In the case of collisions with the 19mer-RNAP template, nearly all of the DNA products were cut by NcoI (Figure 1C, lanes 7–9, SF and TO). Combined with the results from Figure 1D, we conclude that the resolution of this CO replication collision occurred primarily via mRNA takeover, as previously suggested (6). However, if the transcript ended with a chain terminator, a significant amount of the 9.6 kb fragment remained uncut (Figure 1C, lanes 10–12, skip). Thus, replisome skipping could occur if mRNA takeover is unfavorable.

Replication of the 19mer-RNAP, 100mer-RNAP and RNAP array templates showed the formation of stalled replication forks, but the majority of complexes were overcome as indicated by an increase in the formation of the full-length species (Figure 2B and D), as we showed previously (31). However, given the difference in the kinetics of the bypass of different transcription complexes, we wanted to determine whether replisome skipping was utilized in the resolution of more complex replication-transcription collisions. To test this, we digested the replication products with NcoI (Figure 2C). Significantly greater amounts of the NcoI-resistant, 9.6 kbp ‘skip’ product persisted for RNAP array collisions than for collisions with either the 100mer- or 19mer-RNAP (Figure 2E). Furthermore, the CO RNAP array showed higher levels of an uncoupled product (Figure 2C, UC; and F), suggesting that unwinding of the template past the RNAP array without re-initiation of leading-strand synthesis was occurring, as observed previously for replisome bypass of a leading-strand template UV lesion (14).

We assessed the presence of the predicted ssDNA gap by 2D gel electrophoresis of the NcoI-digested 8 min time points (Supplementary Figure S2). The prominent NcoI-resistant 9.6 kb ‘skip’ band of the RNAP array products separated into the 6.9 knt stall product and restart fragments shorter than 2.6 knt upon denaturing gel analysis (Supplementary Figure S2C, purple circle). In contrast, both the 19mer- and the 100mer-RNAP collision products showed little 2.6 knt restart products associated with the faint 9.6 kb product (Supplementary Figure S2A and B, purple circles), suggesting that the primary pathway of resolution for these templates was mRNA takeover. Assessment of mRNA takeover directly demonstrated very low levels of transcript extension for the RNAP array (Figure 3A, lanes 9–12, and Figure 3B and C) and very efficient mRNA takeover for the 19mer-RNAP (Figure 3A, lanes 1–4, and Figure 3B and C). The 100mer-RNAP showed a reduced utilization of mRNA takeover compared to the 19mer-RNAP (Figure 3A, lanes 5–8, and Figure 3B and C), yet the majority of stalled forks from collisions on the 100mer-RNAP template were resolved (Figure 2B and D), suggesting resolution of the CO 100mer may utilize both mechanisms of bypass.

It was shown previously that the RNAP is dislodged from the DNA template in CO collisions with a single RNAP and a short 19mer transcript. Removal of the RNAP coincided with mRNA takeover of the short mRNA transcript (6). We tested whether RNAP removal was also occurring in more complex collisions that are resolved by replisome skipping (Figure 3E and Supplementary Figure S3). 19mer-RNAP and RNAP array-bound templates isolated by gel filtration were either replicated or incubated without replication for 8 min. The reactions were not digested with ScaI until the end of the incubation, thereby allowing replication initiation to proceed continuously. The reaction products were then digested with PvuI, EcoRI and ScaI and the reactions terminated by the addition of EDTA. The terminated reactions were gel filtered in the presence of 0.5 M NaCl to isolate DNA molecules that retained RNAP-complexes. At this concentration of NaCl the ternary complexes stalled by nucleotide starvation remain bound to the DNA, but RNAPs that were displaced during replisome bypass or any RNAPs that re-bound loosely after displacement to other promoters dissociate (47). The excluded volumes were pooled, and the amount of RNAP present in the excluded pools assessed by Western blotting for the presence of the β′ and α subunits of RNAP (Figure 3D, Supplementary Figure S3B and C). Comparison of the amount of RNAP present in the replicated vs. non-replicated samples showed that RNAP was dissociated during replication (Figure 3E). Replication-dependent RNAP dissociation during the CO 19mer collision correlated with levels expected for active RNAP displacement assuming one RNAP per transcript and based on the efficiency of template utilization during replication (as determined by acid-insoluble radioactivity) and the fraction of replicated products that were full length (indicating that bypass of the RNAP had occurred) (Figure 3E and Supplementary Figure S3A). Using this calculation, RNAP dissociation was greater than expected for the CO RNAP array. However, on average, each molecule of RNAP array-template carries more than one RNAP and the replisome may not completely transverse all RNAPs in the array, possibly becoming inactivated. Thus, this result is not surprising and is consistent with the greater fraction of stalled forks remaining after the 8 min incubation for the RNAP array-template compared to the 19mer RNAP–template (Supplementary Figure S3A).

These results demonstrate that the primary mechanism used to resolve CO replication-transcription collisions depended on the complexity of the blockage. Collisions with a single RNAP containing a short transcript were resolved quickly, mainly by mRNA takeover. However, if the RNAP contained a longer transcript or if an RNAP array blocked replication, bypass was slower and increasingly dependent on replisome skipping. Despite the different bypass mechanisms, RNAP displacement appears to be a prerequisite for replisome bypass.

### Priming frequency and replisome stability affect bypass of CO RNAP arrays

**Figure 4.**
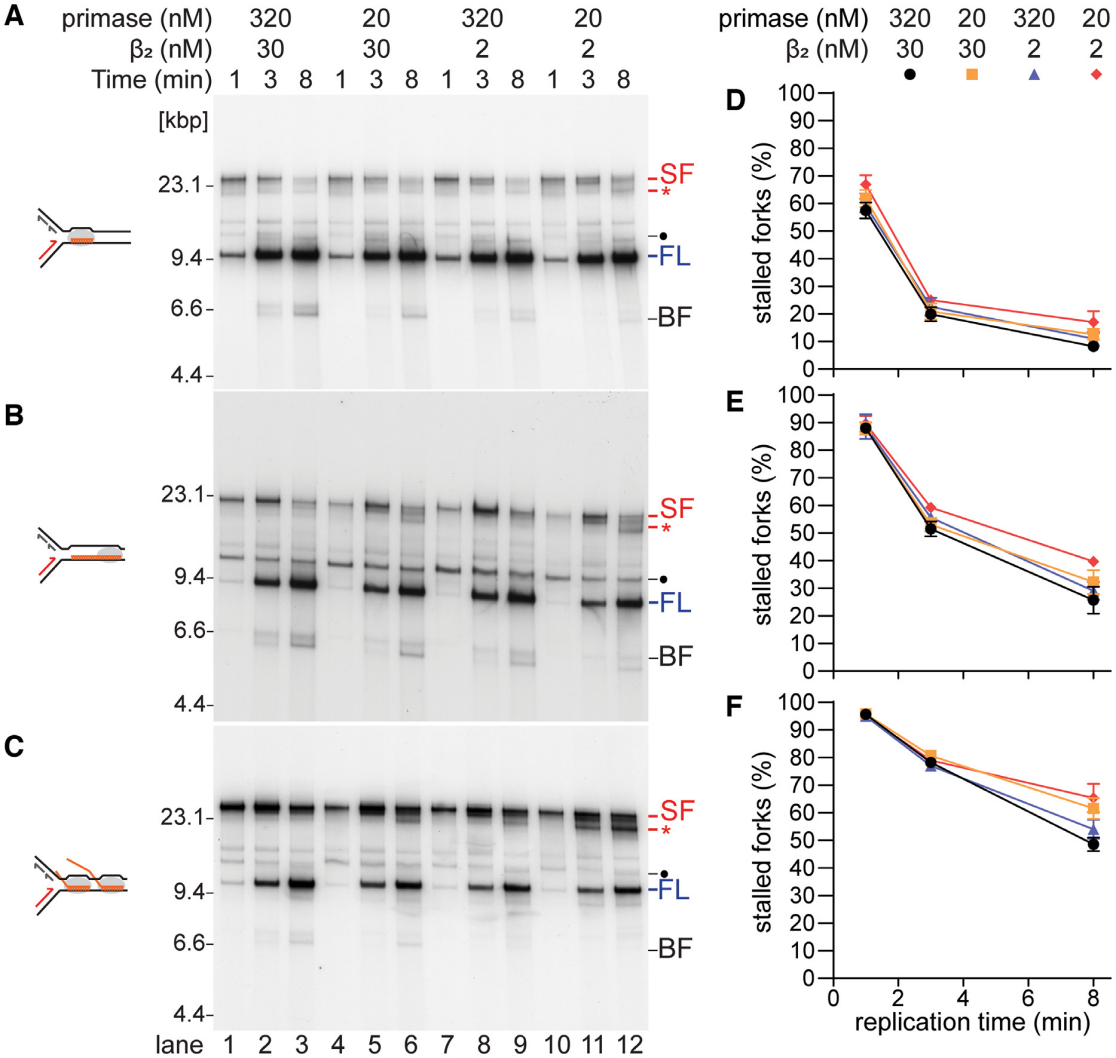
Efficient bypass of CO RNAP arrays is dependent on primase concentration. Native agarose gel analysis of a replication time course of CO replication-transcription collisions with (**A**) the 19mer-RNAP (formed on template CO19), (**B**) the 100mer-RNAP (CO100) and (**C**) the RNAP array (CO100) with differing primase and β concentrations as indicated. Quantification of stalled forks for (**D**) the 19mer RNAP, (**E**) the 100mer RNAP or (**F**) the RNAP array (*n* = 3, mean ± standard deviation). SF, stalled fork; FL, full length; BF, broken fork; gray ovals, RNAP; ‘•’, oriC-independent mRNA extension product (cSDR), most prominent for 19mers that were not extended into 100mers in the 100mer transcription reactions. Free mRNA can be extended by ∼2.6 kb until the Tus-terB barrier is encountered, resulting in a partially duplicated linear DNA fragment of about 13 kb; *, Unwound product resulting from the formation of long ssDNA stretches beyond the RNAP stall by helicase uncoupling and unwinding either without or with inefficient re-priming by DnaG. Note: Both the ‘*’ product shown here and the ‘UC’ product in Figure 2C result from the formation of a ssDNA gap downstream of the RNAP. However, they differ by the length of the ssDNA gap formed downstream of the RNAP. Reduced re-priming at low primase concentrations results in much longer ssDNA gaps that are manifested by a slower migration through the gel of the ‘*’ product compared to that of the ‘UC’ product.

Replisome skipping of leading-strand blockages to DNA replication requires continued procession of the DNA helicase, DnaB and the lagging-strand polymerase downstream of the lesion, albeit at a reduced speed, so that a ss gap can be created to expose DnaG priming sites on the leading-strand template (48). Thus, factors that should affect the efficiency of replisome skipping include reduction of the concentration of the β clamp and of DnaG, which will thereby reduce the processivity of the DNA Polymerase III Holoenzyme (49–51), and the frequency of leading-strand priming (48,52), respectively. Neither of these factors should affect mRNA takeover. To confirm our hypothesis about replisome skipping of complex RNAP blockages, we compared the kinetics of CO RNAP bypass for a single RNAP with a 19mer, whose resolution depended on mRNA takeover, a 100mer-RNAP complex, which is bypassed by a combination of mRNA takeover and RNAP skipping, and an RNAP array, requiring RNAP skipping and re-priming, when the concentrations of β and DnaG were reduced (Figure 4). As expected, we did not observe significant changes for replisome bypass of a single CO 19mer RNAP at either a reduced concentration of primase, the β clamp or both (Figure 4A and D). In contrast, bypass of the CO RNAP array was reduced when either the concentrations of primase or β were reduced. This was most obvious at the 8 min time point (Figure 4C and F). At low primase and β concentrations, the native gels also show a faster moving stalled fork band (Figure 4, *) that increases as a function of the complexity of the replication barrier (compare lane 12 in Figure 4A–C), likely representing uncoupled DNA replication, where the template DNA is unwound beyond the RNAP complexes, but re-priming has not occurred. Thus, both replisome stability (β clamp) and the kinetics of re-priming (primase) affect bypass of CO RNAP arrays. Replication bypass of a 100mer RNAP was moderately affected at reduced primase concentrations and was further exacerbated by also reducing the β concentration (Figure 4B and E), confirming that bypass of a 100mer RNAP occurs by both replisome skipping in addition to mRNA takeover (Figure 3).

### UvrD, Mfd and Rep promote continuous leading-strand synthesis

**Figure 5.**
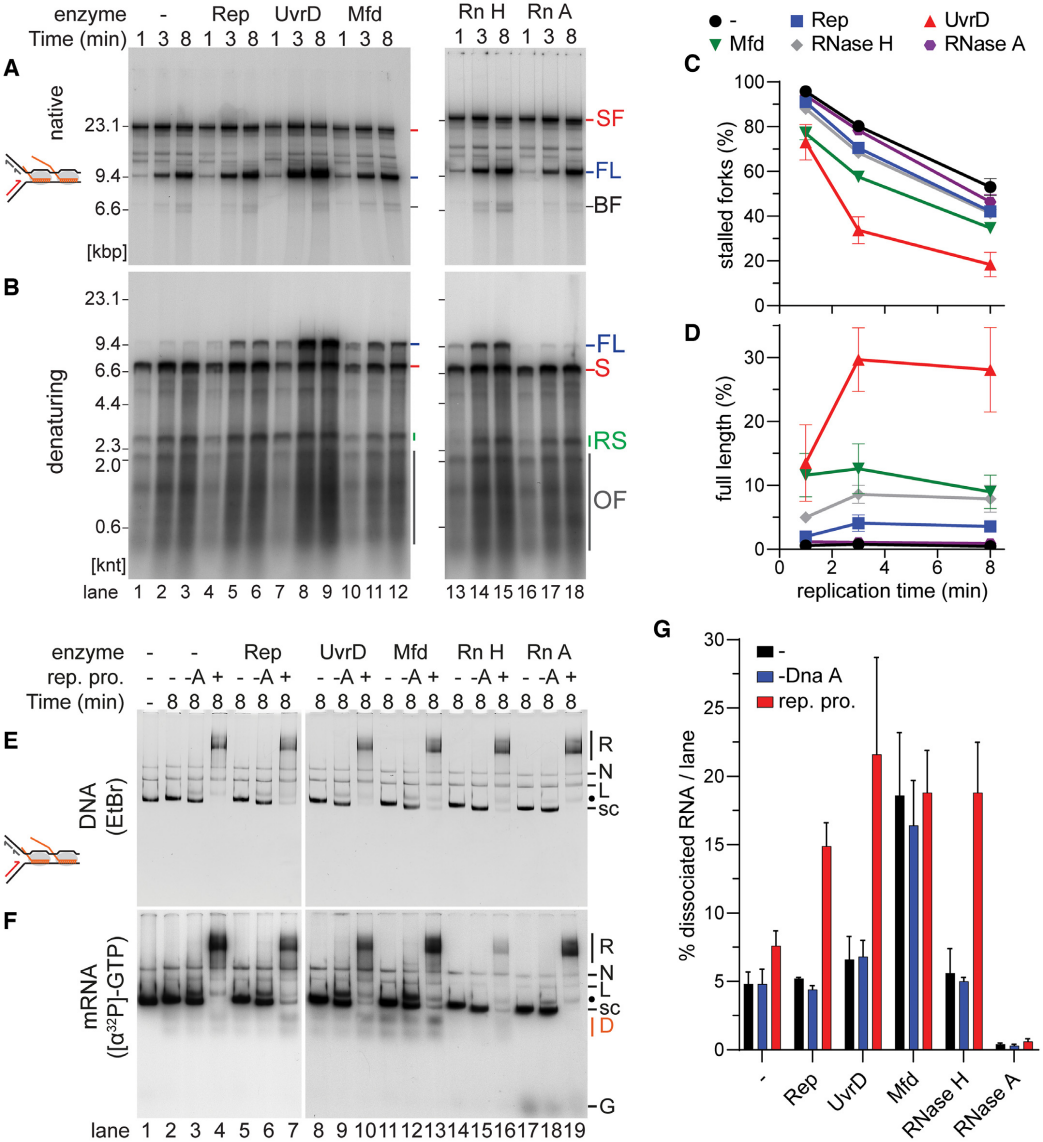
Bypass of CO RNAP arrays can be promoted by additional factors. Native (**A**) and denaturing (**B**) agarose gels of products in replication time courses of CO replication collisions with RNAP arrays (on template CO100) after the indicated factors were added for 10 min prior to the initiation of replication. Final concentrations were 100 nM Rep (lanes 4–6), 100 nM UvrD (lanes 7–9), 500 nM Mfd (lanes 10–12), 0.1 U/μl RNase H (lanes 13–15), or 20 μM RNase A (lanes 16–18). Quantification of (**C**) stalled forks or (**D**) full length products (*n* = 3, mean ± standard deviation). Native agarose gel of replication reaction products (**E**) stained with ethidium bromide or (**F**) visualized by autoradiography of [α-^32^P]GMP-labeled mRNA. Pooled gel filtered DNA-RNAP complexes (lane 1) were incubated for 10 min with the indicated factors (same concentrations as in panel A). Incubation was continued for another 8 min with the omission of any replication proteins (–) or with either the full complement of replication proteins (+) or with DnaA omitted (–A). Reactions were terminated by the addition of 30 mM EDTA without any restriction enzyme digestion. (**G**) Quantification of the fraction of displaced mRNA products (*n* = 3, mean ± standard deviation). SF, stalled fork; FL, full length, BF, broken fork; S, leading-strand stall product; RS, leading-strand restart products; OF, Okazaki fragments; R, replicated; N, nicked; L, linear; s.c., supercoiled; D, displaced mRNA; G, [α-32P]GTP; Rn H, RNase H; Rn A, RNase A; rep. pro.; replication proteins. Note that the position of the oriC-independent mRNA extension product denoted by ‘•’ is different on the gels shown in panels E and F compared to the gels shown in Figure 4A–C because in the latter case the DNA products were digested with PvuI and EcoRI, whereas in the former case they were not.

There are multiple mechanisms that are thought to resolve replication-transcription collisions *in vivo*. For example, the helicases Rep and UvrD and the translocase Mfd can promote replisome bypass at head-on replication-transcription collisions (22,31,53). Although CO arrays were eventually overcome without assistance (Figure 2) (31), we tested whether any of these accessory proteins could improve the efficiency of CO RNAP bypass as well. We incubated RNAP array-DNA templates either without any accessory protein or with Rep, UvrD, or Mfd for 10 min prior to the initiation of replication. Analysis of the products of the replication time course on native gels showed a slight improvement in replisome bypass in the presence of Rep compared to the no protein control. Stalled fork levels were further reduced in the presence of Mfd and, most prominently, in the presence of UvrD (Figure 5A and C). Analysis of these products on denaturing agarose gels showed increased formation of full-length products. Again, UvrD had the greatest effect, followed by Mfd and Rep (Figure 5B and D). We repeated the experiment without the 10 min pre-incubation of the factors with RNAP-DNA templates (Supplementary Figure S4). In this case, Mfd no longer promoted significant replisome bypass, whereas Rep and UvrD showed similar kinetics as with the pre-incubation (compare Figure 5C and Supplementary Figure S4D). We monitored the association of [α-^32^P]GMP-labeled mRNA with ethidium bromide stained DNA (Figure 5E and F) after a 10 min incubation with the indicated enzyme and either a further incubation of 8 min without any replication enzymes, with all replication enzymes except DnaA, or with the full set of replication enzymes. To maintain protein-DNA complexes formed during the experiment, the reactions were not digested with restriction enzymes. Control replication reactions where the nascent DNA was labeled with [α-^32^P]dAMP showed that these alterations did not significantly affect the outcomes of the replication reactions (compare Supplementary Figure S5A and Figure 5B). Furthermore, incubation with the enzymes did not affect the integrity of the mRNA transcripts, nor did any of the enzymes cause an increase in mRNA takeover (Supplementary Figure S5B). Low levels of mRNA extension that are observed are likely the result of *oriC*-independent extension of transcripts lacking the RNAP (Supplementary Figures S5A and B, compare lanes –A and +), where the presence of a free 3′-OH group allows their extension by Pol III* (31). Quantification of mRNA dissociation showed an effect of Mfd even in the absence of replication proteins. In contrast, Rep and UvrD displaced mRNA only under conditions where DNA replication occurred (Figure 5G, +rep. pro.). These results suggest that unlike Mfd, Rep and UvrD require an active replication fork collision to promote displacement, as shown previously (22).

Similar to an RNAP array, UvrD was also able to reduce fork stalling upon collisions with an R-loop array (Supplementary Figures S6A and C). This coincided with an increase in continuous full-length, leading- strand products (Supplementary Figures S6B and D). The presence of UvrD resulted in increased levels of RNA dissociation from the DNA (Supplementary Figure S6H, lane 8, and Supplementary Figure S6I), coinciding with a reduction of the signal of the labeled RNA co-migrating with the replicated, slow-moving DNA species (Supplementary Figure S6H, lane 8). UvrD was equally efficient at promoting replisome bypass when the 10 min preincubation was omitted and UvrD was added at the time of replication initiation (Supplementary Figure S6E and F). However unlike in the case of the RNAP array, the addition of UvrD also increased levels of dissociated RNA molecules compared to the untreated control in the absence of any replication proteins (Supplementary Figure S6H, lanes 2 and 4, and Supplementary Figure S6I). In contrast, Rep and Mfd had no effect compared to the untreated controls with or without the 10 min preincubation (Supplementary Figure S6). Thus, UvrD can remove R-loops from DNA when an RNAP is absent, thereby suppressing replication fork stalling by clearing at least some of the obstacles before they are encountered by a replisome.

### The length on the template occupied by the obstacle is a major determinant of its impact on CO collision bypass

In our previous work, we showed that RNAP-free R-loops on the leading-strand template were a minor impediment to replication fork progression (31). We examined the role of the transcripts on the CO replication-transcription collisions by treating the DNA–RNAP complex arrays with either RNase H or RNase A prior to replication initiation. In the presence of RNase H, stalled forks were reduced to levels similar to those in the presence of Rep (Figure 5A and C). Similarly, the amount of full-length product formed was increased (Figure 5B and D), suggesting that treatment with RNase H facilitated replisome bypass likely by reducing the complexity/extent of the RNAP array upon replisome collisions (see below). However, given the presence of multiple RNAPs on the template, the removal of the R-loop portion of the obstacle, which is most likely associated with only the last trailing RNAP, only resulted in a modest improvement in replisome bypass. Quantification of the restart products (RS) present in Figure 5B (lanes 1–3), adjusted for the DnaA-independent product evident in lane 1 that has the same electrophoretic mobility as the restart products (see Supplementary Figure S5A), showed that a majority of the CO RNAP arrays were clearly bypassed by replisome skipping [at the 8 min time point restart products increased to 30% as stalled forks decreased from 95% at the 1 min time point to 55% at the 8 min timepoint (Figure 5A and C)]. In contrast, RNase A had little to no effect and its replication profiles resembled those of the no enzyme control (Figure 5A–D). Examination of the effect of the RNases on the [α-^32^P]GMP-labeled mRNA transcript revealed that degradation (Supplementary Figure S5B, lanes 14–16), as well as displacement (Figure 5E–G, lanes 13–15), of the transcripts by RNase H was dependent on DNA replication. It is possible that RNase H can only degrade the mRNA/R-loop after the RNAP complex has been displaced by a replication fork. RNase A treatment degraded the transcripts to a mixture of 18–20 nt-long fragments, about the length of the footprint of the arrested RNAP, and longer fragments presumably representing hybridized portions of the RNA in R-loops (Supplementary Figure S5B, lanes 17–19) (54). This is different from what we observed previously with R-loops formed by the degradation of RNAPs, where radioactive R-loops dissociated from the DNA upon RNase H, but not RNase A treatment (31), suggesting that removal of the RNAP allows for more extensive RNA–DNA hybrid formation. Mapping experiments showed that leading-strand synthesis stalled at the transcription start site, even for the RNAP array (Supplementary Figure S7C, lanes 1 and 4). No product was formed that would indicate a direct collision of the polymerase with the RNAP. After treatment with RNase H, leading strands were elongated by about the length expected for a direct collision with the RNAP (Supplementary Figure S7C, lanes 2 and 5). In contrast, RNase A treatment promoted extension to a much lesser degree (Supplementary Figure S7C, lanes 3 and 6). Stalling products formed independent of template topology, excluding the possibility that replication-dependent supercoiling created a topological strain between the replisome and the RNAP (Supplementary Figure S7C, linearized). We conclude that the transcripts formed RNA–DNA hybrids, likely stabilized by the RNAP, even on linearized DNA, unless processed by RNases. However, the mRNA transcripts were likely only partially hybridized, as both RNases could attack the transcript at multiple sites, leading to the degradation patterns observed in Supplementary Figure S5B.

**Figure 6.**
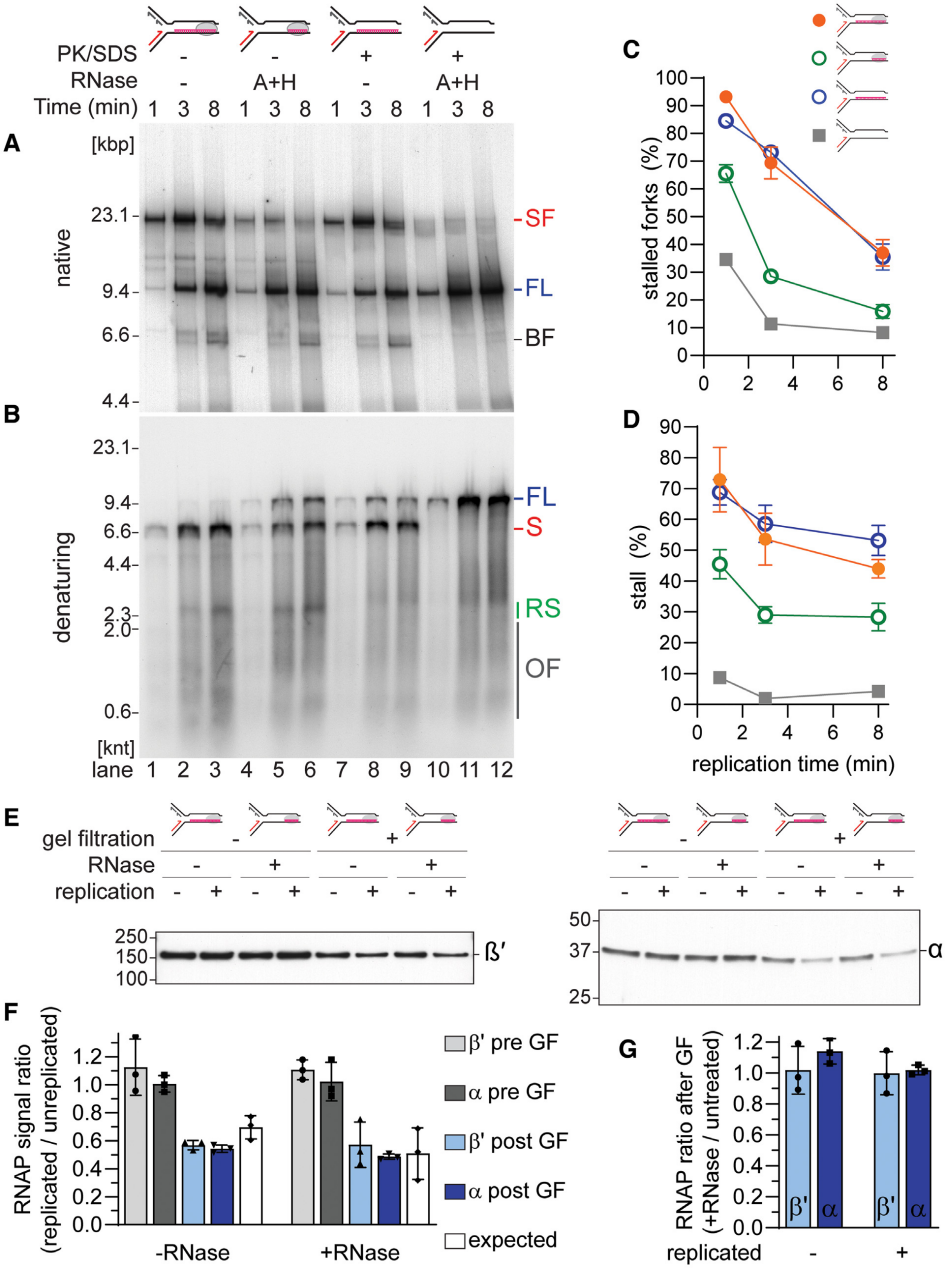
Longer R-loops dictate replisome stalling in co-directional replication-transcription collisions. Native (**A**) and denaturing (**B**) agarose gels of products in replication time courses of CO replication collisions with a single RNAP and a 100mer transcript (formed on template CO100, lanes 1–6) or a 100mer R-loop (CO100, lanes 7–12) subsequent to a 10 min incubation of the templates either without RNase, or with 0.1 U/μl RNase H and 20 μM RNase A (A + H). Quantification of (**C**) stalled forks and (**D**) stall products (*n* = 3, mean ± standard deviation). (**E**) Western blot analysis of β′ (left) and α (right) subunits of CO replication collisions with a single RNAP and a 100mer transcript (formed on template CO100) with or without replication proteins and RNase treatment, and before or after gel filtration in high salt. (**F**) Ratios of β′ and α RNAP subunit signal intensities from Western blots of replicated versus unreplicated reactions before (gray bars) and after (blue bars) gel filtration in high salt compared to the RNAP subunit ratio predicted for active RNAP dissociation by replisome bypass (white bar) (*n* = 3, mean ± standard deviation). (**G**) Ratios of β′ (light blue) and α (blue) RNAP subunit signal intensities after gel filtration in high salt from Western blots of RNase treated versus untreated samples (*n* = 3, mean ± standard deviation). SF, stalled forks; FL, full length; BF, broken fork; S, leading-strand stall product; RS, leading-strand restart products; OF, Okazaki fragments.

To examine the contribution of the transcripts and the RNAP on replication stalling individually, we generated a CO 100mer RNAP transcript and incorporated a chain terminator to prevent *oriC*-independent extension of R-loops later in the replication stage of the reactions (31). The transcription reaction was divided in half and the RNAP was either left intact or degraded with Proteinase K and SDS before replication templates were isolated by gel filtration. Pooled replication templates were either treated or not treated with RNase H and RNase A prior to replication initiation to degrade R-loops and all accessible un-hybridized RNA, respectively. The intact transcription complex with the CO 100mer mRNA generated similar levels of stalled replication forks as the 100mer R-loop itself (Figure 6A, compare lanes 1–3 and 7–9, and Figure 6C). Note that the incorporation of the chain terminator increases replisome stalling, as we showed in Figure 1C for a single RNAP with a 19mer and also for the single RNAP with a 100mer transcript (compare Figure 2D and 6C). Denaturing gel electrophoresis revealed an increase in the amount of full-length product generated in the reactions with the R-loop template (Figure 6B, lanes 7-9). This is likely caused by low levels of R-loop dissociation during gel filtration (31). Overall, the full transcription complex and the 100mer R-loop posed an equally efficient barrier to the replisome (Figure 6D). Because the mRNA in the RNAP-transcript complexes and the RNA in the R-loops were capped with a chain terminator, neither replication bypass via mRNA takeover nor *oriC*-independent extension of the R-loops was possible in these reactions. Thus, the appearance of the restart product over time indicated replisome bypass via replisome skipping. RNase treatment of the R-loop template prevented replication fork stalling (Figure 6A, compare lanes 10–12 to lanes 7–9) and the formation of the stall product (Figure 6B, compare lanes 10–12 to lanes 7–9), suggesting that the both the RNAP and the R-loop had been removed from these templates by the SDS/Proteinase K and RNase treatments, respectively. Instead, in the absence of a replication obstacle, replication proceeded uninterrupted and only a full-length product was generated (Figure 6B, lanes 10–12). However, when the R-loop portion of the transcription complex was degraded and only the single RNAP was left behind, replication stalling was reduced (Figure 6A lanes 4–6 and 6C), suggesting a single RNAP without a trailing transcript was less of a replication obstacle than a long R-loop when encountered co-directionally with replication fork movement. Similar to the R-loop template alone, replication of the RNAP-only templates generated increased levels of full-length replication products (Figure 6B, lanes 4–6).

To confirm that the reduction in stalled replication forks was not simply a result of increased dissociation of the transcript-free RNAPs from the DNA template prior to an encounter with an active replication fork, we measured RNAP association with the templates by Western blotting with and without RNase treatment (Figure 6E). After gel filtration in the presence of 0.5 M NaCl, replicated samples showed reduced levels of RNAP compared to unreplicated samples. The ratio of the RNAP subunits present for replicated and unreplicated reactions was close to the value expected for active replisome displacement based on full-length product formation and template utilization in the individual experiments (Figure 6F, determined as described for Figure 3), suggesting only replication-dependent RNAP removal occurred. Importantly, RNase treatment did not cause increased RNAP dissociation from either the unreplicated or replicated samples (Figure 6G). Therefore, a single RNAP without a transcript is more easily bypassed by the replisome compared to the whole transcription complex or the R-loop alone. Conversely, this means that longer R-loops are the main replication obstacle when encountered co-directionally. Combined with the fact that RNase H treatment of the RNAP array template only resulted in a moderate improvement in replisome bypass (Figure 5A–D), these data suggest that the main determinant for replication stalling is the overall length occupied on the template, rather than the type of the obstacle: a longer RNAP-free R-loop can be just as problematic as an RNAP array.

## DISCUSSION

**Figure 7.**
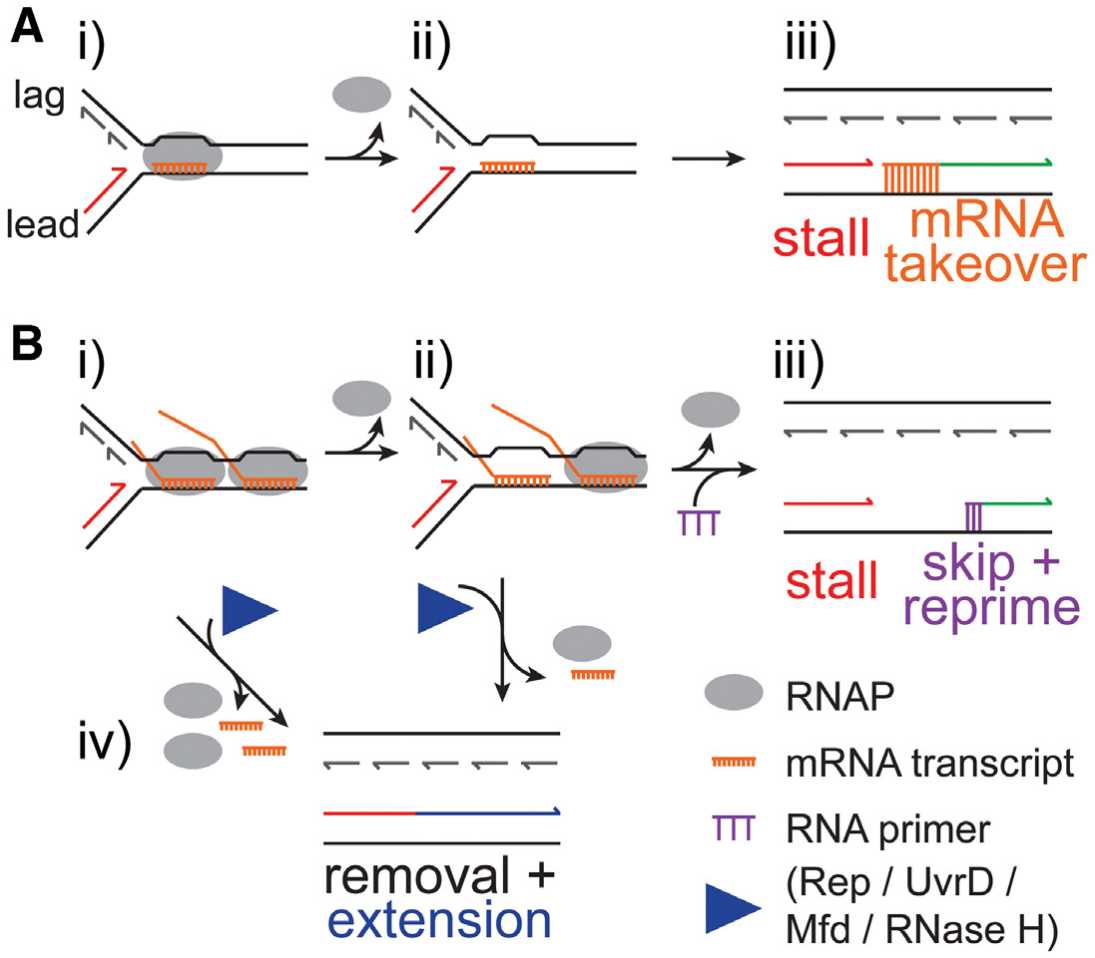
Mechanisms of Bypass of CO RNAP-Transcription Complexes. (**A**) Bypass of a single CO RNAP with a short transcript. (i) Replisome encounter with a CO 19mer (ii) leads to RNAP dissociation. (iii) The 3′ end of the nascent mRNA transcript is extended by the replisome (‘mRNA takeover’), resulting in a discontinuous leading strand containing a very short ssDNA gap. (**B**) Bypass of co-directional RNAP arrays. (i) Replisome encounter with a CO RNAP array. (ii) Incomplete displacement of the RNAP array by the replisome preserves stalled replication forks. (iii) Removal of the remaining RNAP(s) followed by synthesis of a new leading strand primer downstream of the collision allows the formation of a discontinuous leading strand with a longer ssDNA gap. The nascent mRNA transcripts (not shown) may remain bound to the template, requiring removal prior to post-replicative filling of the ssDNA gap. (iv) RNAP removal can be promoted or facilitated by various factors resulting in the formation of a continuous leading strand.

A CO replisome collision with a single RNAP complex was only a minor block to replication fork progression (Figures 1C and 2B), as demonstrated previously (6). Replisomes could also bypass an RNAP array (Figure 2B) (31), although the primary mechanism utilized in these two cases differed (Figure 7). The simplest mechanism by which a replisome could overcome a collision with an RNAP would be the direct removal of the RNAP and the mRNA by the replisome itself. This pathway would be evident in our experiments by the formation of continuous full-length leading strands that could be visualized by denaturing agarose gel electrophoresis. However, we did not observe significant formation of such full-length products for replisome encounters with RNAPs (e.g. Figures 5B, 6B and (31)). Because the replicative helicase DnaB translocates on the lagging-strand template of the replication fork, it will not encounter and remove the mRNA/R-loop that is hybridized to the leading-strand template in a CO collision. Instead, short transcripts were taken over by the replisome to continue leading-strand synthesis (Figures 1D and 3A) (6). Leading-strand synthesis stopped at the transcription start site rather than upon contact with the stalled RNAP, suggesting that (at least the 5′-end of) the mRNA hybridized with the negatively supercoiled DNA upstream of the RNAP (Supplementary Figure S7). It is possible that polymerases and RNAPs collide directly if the mRNA transcript is less prone to forming R-loops or if RNA hybridization is reduced *in vivo*, where transcripts would be bound by ribosomes. However, in either case, bypass via mRNA takeover should leave only a short ssDNA gap, similar to those created during Okazaki fragment synthesis. Resolution of collisions with promoter-proximal RNAPs by mRNA takeover is likely favorable, as the loss of an early mRNA transcript is still preferable over the formation of a long ssDNA gap.

Replisome bypass of RNAP arrays depended primarily on re-priming downstream of the RNAP array and resumption of DNA synthesis, leaving a longer nascent leading-strand gap (Figure 2C, Supplementary Figures S2C and S3). The same mechanism is utilized by the replisome to bypass different types of base damage on the leading-strand template (14,48). This dependency on re-priming is further supported by reduced bypass of CO RNAP arrays, but not CO 19mer-RNAPs, at reduced primase concentrations (Figure 4). A large proportion of the NcoI-digested reaction products revealed significant amounts of an uncoupled product for the RNAP array (Figure 2C and Supplementary Figure S2C, UC). Because RNAP stalling is maintained by omitting CTP and UTP in the replication reactions, priming frequency is reduced (31). It is likely that in the presence of all four NTPs, the formation of this uncoupled product would be reduced in favor of greater amounts of the skip product.

Our data suggests that the RNAP complexes are dislodged during replication bypass (Figure 3E, Supplementary Figure S3 and S6F). Direct RNAP removal has been shown previously (6). These results are in contrast to previous work where an *Escherichia coli* RNAP complex remained bound to the DNA after being bypassed by a bacteriophage T4 replisome (55). The greater than expected RNAP displacement for CO array collisions (Figure 3E) support work that showed replisome breakdown at highly transcribed co-directional genes (4). Thus, RNAP displacement is stochastic, reducing the likelihood of replisome bypass with each additional RNAP present in any particular blockage. Our data could not determine if the replisome collapses at more complex RNAP collisions or if it remains active until additional factors, such as helicases or transcription coupled repair factors lead to removal or reactivation of the RNAP. However, in any case, RNAP removal is a prerequisite for bypass by both mRNA takeover and replisome skipping. Because replisome skipping was also observed for transcripts that lacked a 3′-OH (Figure 1C), in sum, these observations suggest that replisome skipping is a dominant mechanism *in vivo*, used not only for replication past DNA lesions, but also for the bypass of stalled or even transcribing CO RNAPs.

Head-on collisions require further processing to allow replisome progression (31,53). However, it has been shown that replisomes also frequently collapse at highly transcribed genes oriented CO with replication fork progression *in vivo* (4). Of the two accessory helicases tested, UvrD was quite efficient in promoting replication bypass of CO RNAP, as well as R-loop arrays in favor of continuous leading-strand synthesis (Figure 5 and Supplementary Figure S6), suggesting that CO RNAP and R-loop bypass could also be promoted actively *in vivo*. mRNA displacement by Rep and UvrD was dependent on active DNA replication (Figure 5G) (22), possibly because the RNAP transcription complex alone does not provide access to a long enough stretch of ssDNA for the helicases to bind. This would prevent unrestricted removal of actively transcribing RNAPs by these helicases *in vivo*. However, an approaching replication fork could generate stretches of ssDNA that allow Rep and UvrD to be loaded. We attribute the greater efficiency of UvrD in resolving RNAP-replisome collisions (Figure 5) to its direct recruitment to RNAPs via its C-terminal tail (56). Rep localizes directly to the replisome via an interaction with the helicase DnaB (19,57) and may quickly be moved past the RNAPs by uncoupling of the helicase. The activity of Mfd to dissociate mRNA was replication independent (Figure 5G). Mfd removes only stalled RNAPs (24,58). Given the nucleotide restriction used in our assays, RNAP complexes were either stalled or backtracked and therefore subject to removal by Mfd (58). The reduced promotion of replisome bypass when the incubation step is omitted (Supplementary Figure S4) was likely because of the slow catalytic activity of Mfd (58). However, in the absence of an RNAP, a naked R-loop becomes a substrate for UvrD, but not Rep or Mfd. Because UvrD is a potent helicase on RNA-DNA substrates (59) and was able to displace naked R-loops from the DNA template even in the absence of DNA replication (Supplementary Figure S6G), UvrD may monitor the genome and remove R-loops within cells.

Our results further elucidate the effects of R-loops in CO replication-transcription collisions, suggesting that the length of R-loops formed behind the RNAP determines the severity of the CO collision to a similar degree as the total number of RNAPs in the array. RNase H treatment (together with RNase A) of CO RNAP collisions led to a reduction in replisome stalling that was reflective of the length by which the obstacle was reduced (Figures 5 and 6). Thus, the total distance of an obstacle that the replisome needs to overcome is a strong determinant of the severity of the replication blockage (compare the RNAP array in Figure 5A and C to the CO 100mer R-loop in Figure 6A and C). It is unclear if CO R-loops delay replisome bypass by acting mainly as an obstacle to the leading-strand polymerase, which travels along the leading-strand template to which the R-loop is hybridized, or if the CO R-loops present an additional obstacle to the progression of the replicative helicase past the RNAP(s). Little to no R-loop induced genome instability has been observed for CO collisions in bacteria (32), suggesting that the replicative helicase can translocate unobstructed along the R-loop-free lagging-strand template (Figure 7). Nevertheless, despite this theoretically simple mechanism for bypass, an R-loop of moderate length (100 nt) was an obstacle to replication fork progression in our study. Thus, it is certainly possible that R-loops of more than 1 kb could have an even greater impact on replisome progression (60,61). It is also conceivable that short stretches of transcripts that are not protein-bound could still (temporarily) hybridize to the DNA and act as an obstacle to the leading-strand polymerase. In rare events, or in R-loop prone mutant backgrounds, longer R-loops could wrap around the lagging-strand template before hybridizing back to the leading-strand template. The introduction of such ‘knots’ could impede helicase progression.

RNase H treatment did however increase the formation of continuous leading strands (Figures 5B and D and 6B and D). Removal of the transcript from an RNAP (Figure 6) modeled a more promoter-proximal transcription complex. Thus, replisome bypass is more efficient for RNAPs with short transcripts, likely by enabling the R-loops to be removed via the strand displacement activity of the leading-strand polymerase (62). This is in agreement with the inability of short RNAP-free 19mer R-loops, but not 100mer R-loops, to cause the formation of leading-strand gaps (31). Thus, even though there may be a – albeit reduced – replication delay because of the presence of the remaining R-loop-free RNAP, the outcome of RNase H treatment is a nascent leading strand that does not require any post-replicative processing, potentially avoiding the toxicity reported for CO replication-transcription collisions (13).

## DATA AVAILABILITY

Plasmid DNA, sequences, and *E. coli* strains generated in this study will be supplied upon request.

## Supplementary Material

gkab760_Supplemental_File
