# Supplementary material for: Bypass of complex co-directional replication-transcription collisions by replisome skipping
Source: Nucleic Acids Res. 2021 Sep 1;49(17):9870–85. doi: 10.1093/nar/gkab760 (PMC8464059; doi:10.1093/nar/gkab760)
Supplement: gkab760_Supplemental_File [file gkab760_supplemental_file.pdf]

SUPPLEMENTARY INFORMATION

Bypass of Complex Co-directional Replication-Transcription Collisions by Replisome Skipping

Jan-Gert Brüning and Kenneth J. Marians*

Molecular Biology Program, Memorial Sloan Kettering Cancer Center, 1275 York Avenue, New York, NY 10065, USA

*correspondence: Tel. (212) 639-5890; e-mail. kmarians@sloankettering.edu

Supplementary Figures 1-7 and Figure Legends

Supplementary Figure 1

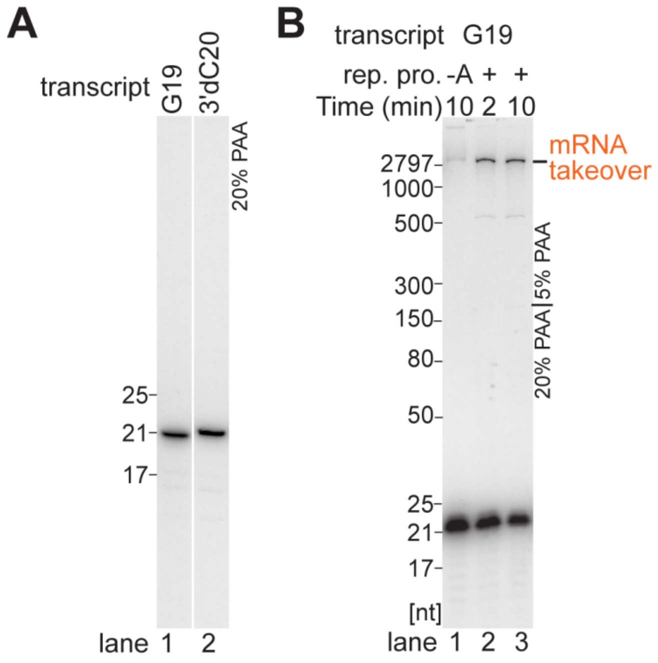

Supplementary Figure 1. mRNA takeover controls (related to Fig. 1).
(A) A single 3′-dCTP chain terminator is incorporated by RNAP into the transcripts. Transcription reaction mixtures containing [α-^{32}P]GTP with the CO_{19} template either without (G19) or with (3'dC20) 3′-dCTP were analyzed by electrophoresis through 20% 7M urea polyacrylamide gels.
(B) mRNA takeover requires replication initiation from *oriC*. Replication reactions containing the 19mer-RNAP template where the mRNA had been labeled with [α-^{32}P]GMP were incubated for the indicated times with either the full complement of replication proteins (rep. pro.,+) or with DnaA omitted (-A) and analyzed by electrophoresis through a composite 7M urea 5%/20% polyacrylamide gel (n=3).

Supplementary Figure 2

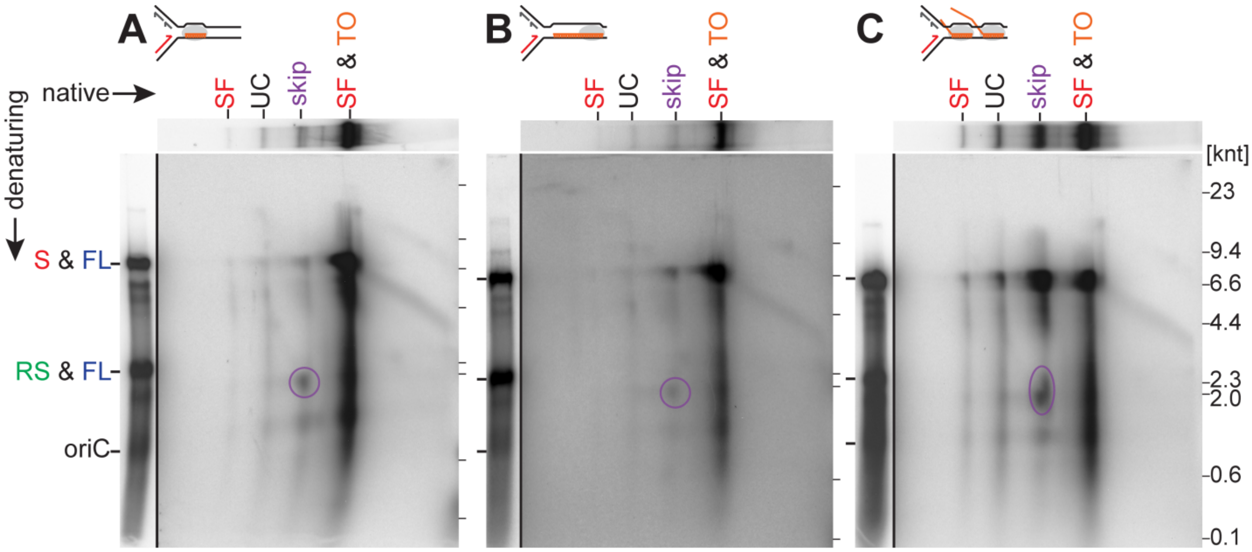

Supplementary Figure 2. Replisome re-priming occurs during replisome bypass of RNAP arrays (related to Fig. 2).
2D-gel analysis of NcoI-digested replication reaction products at the 8 min time point from Figure 2C: (A), 19mer RNAP; (B), 100mer RNAP; (C), RNAP array. Replisome skip products are circled in purple (n=2). S, stalled nascent leading strand; FL, full-length product; RS, restarted leading-strand products; *oriC*, 0.8 kb EcoRI-PvuI fragment carrying *oriC* and the terminated counterclockwise-moving fork; SF, stalled fork; UC, uncoupled product (14); skip, NcoI-resistant material resulting from replisome skipping; TO, mRNA takeover product; grey ovals, RNAP.

Supplementary Figure 3

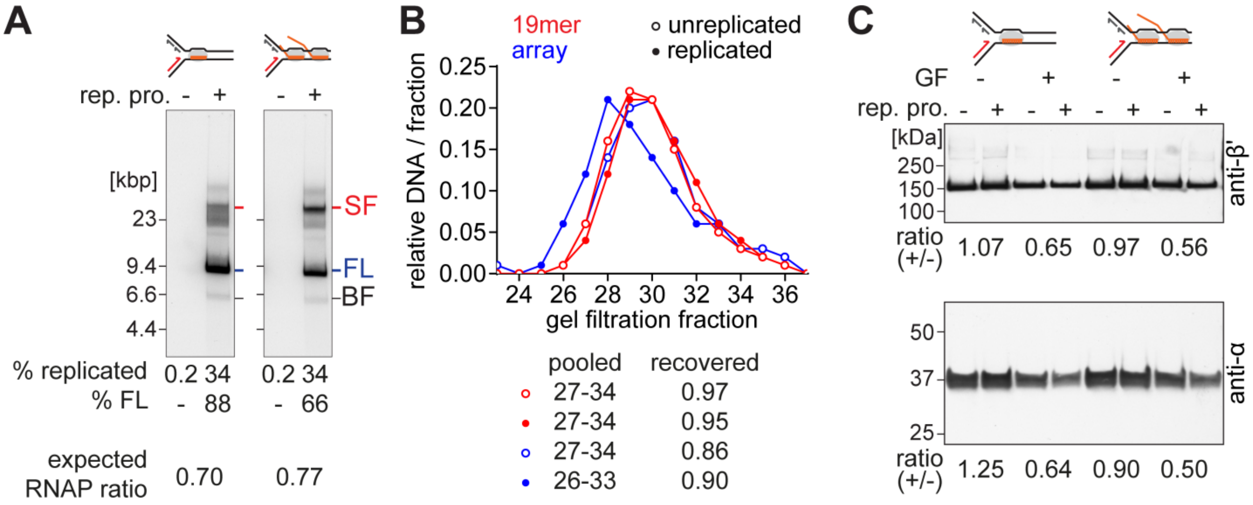

Supplementary Figure 3. Replisome bypass of RNAP obstacles involves RNAP displacement (related to Fig. 2).

(A) Native agarose gel of products of replication reactions of CO replication collisions with a single RNAP with a 19mer transcript (formed on template CO_{19}) or an RNAP array (CO_{100}) without or with replication proteins. Estimated template utilization (% replicated) was calculated from the incorporation of radioactive precursor into acid-insoluble product and the fraction of full length products (% FL) from the replication product profiles on the native gels as described in the methods section. The expected RNAP ratio is calculated by subtraction from 1 of the product of the replicated templates and levels of full length products.

(B) Elution profiles of after gel filtration in the presence of 0.5 M NaCl for each of the four different replication reactions shown in panel A. Relative amounts of DNA were based on DNA readings on a Nanodrop for unreplicated samples using fraction number 22 as a blank, or using the relative amounts of measured radioactivity for the individual fractions of replicated samples. DNA recovery is shown for the indicated pooled fractions.

(C) Western blot analysis of the β' and α RNAP subunits of the different replication reactions before and after gel filtration in the presence of 0.5 M NaCl. Ratios are calculated as the fraction of the subunit intensities of the replicated versus the unreplicated reactions. SF, stalled fork; FL, full length; BF, broken fork, GF, gel filtration; rep. pro., replication proteins; grey ovals, RNAP.

Supplementary Figure 4

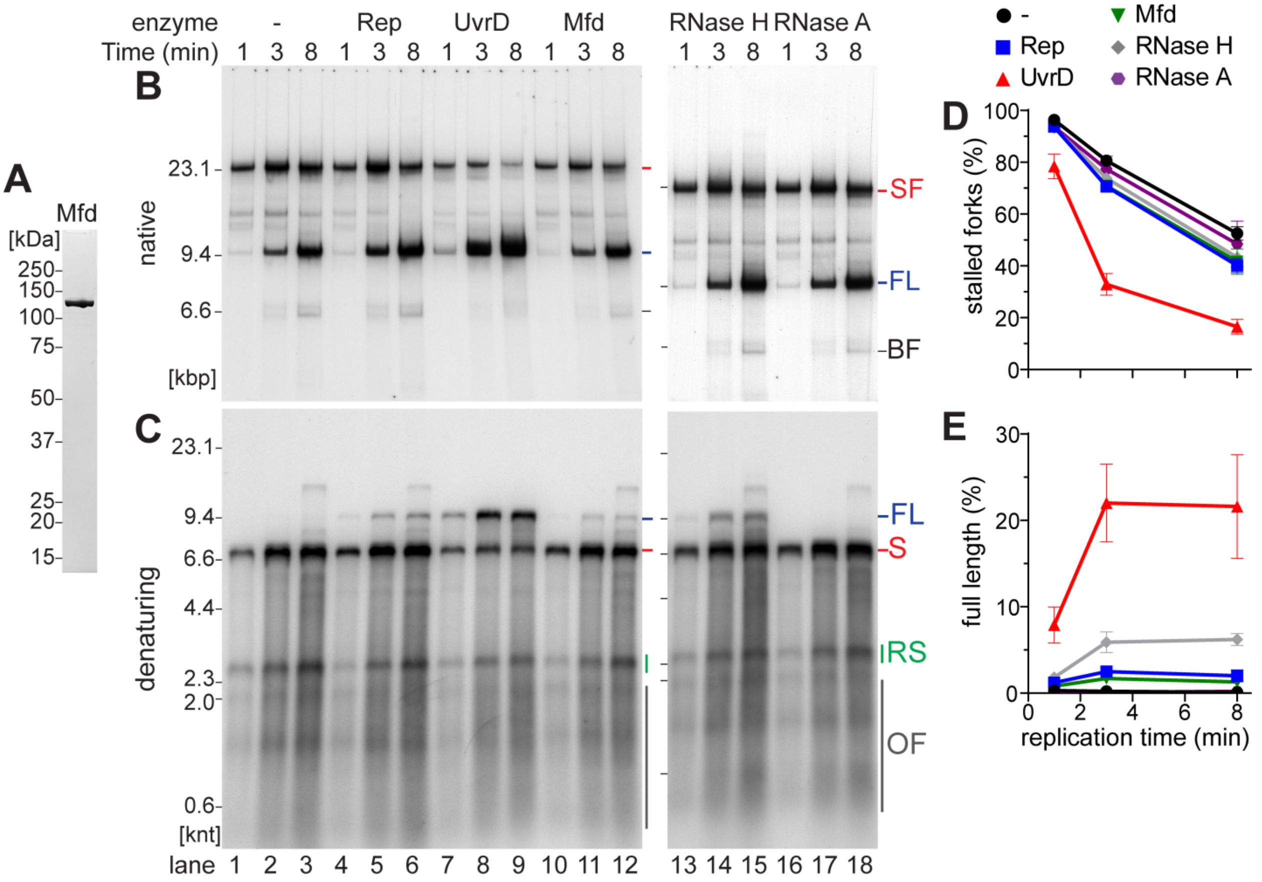

Supplementary Figure 4. Mfd requires pre-incubation to promote efficient replisome bypass of CO RNAP arrays (related to Fig. 5).
(A) Purified Mfd analyzed by 8% SDS-PAGE.
(B) Native and (C) denaturing agarose gels of products in replication time courses of CO replication collisions with RNAP arrays (on template CO_{100}). The indicated factors were added together with replication proteins without any prior incubation. Final concentrations were as in Figure 5.
Quantification of (D) stalled forks or (E) full length products (n=3, mean ± standard deviation).
SF, stalled fork; FL, full length; BF, broken fork; S, leading-strand stall product; RS, leading-strand restart products; OF, Okazaki fragments.

Supplementary Figure 5

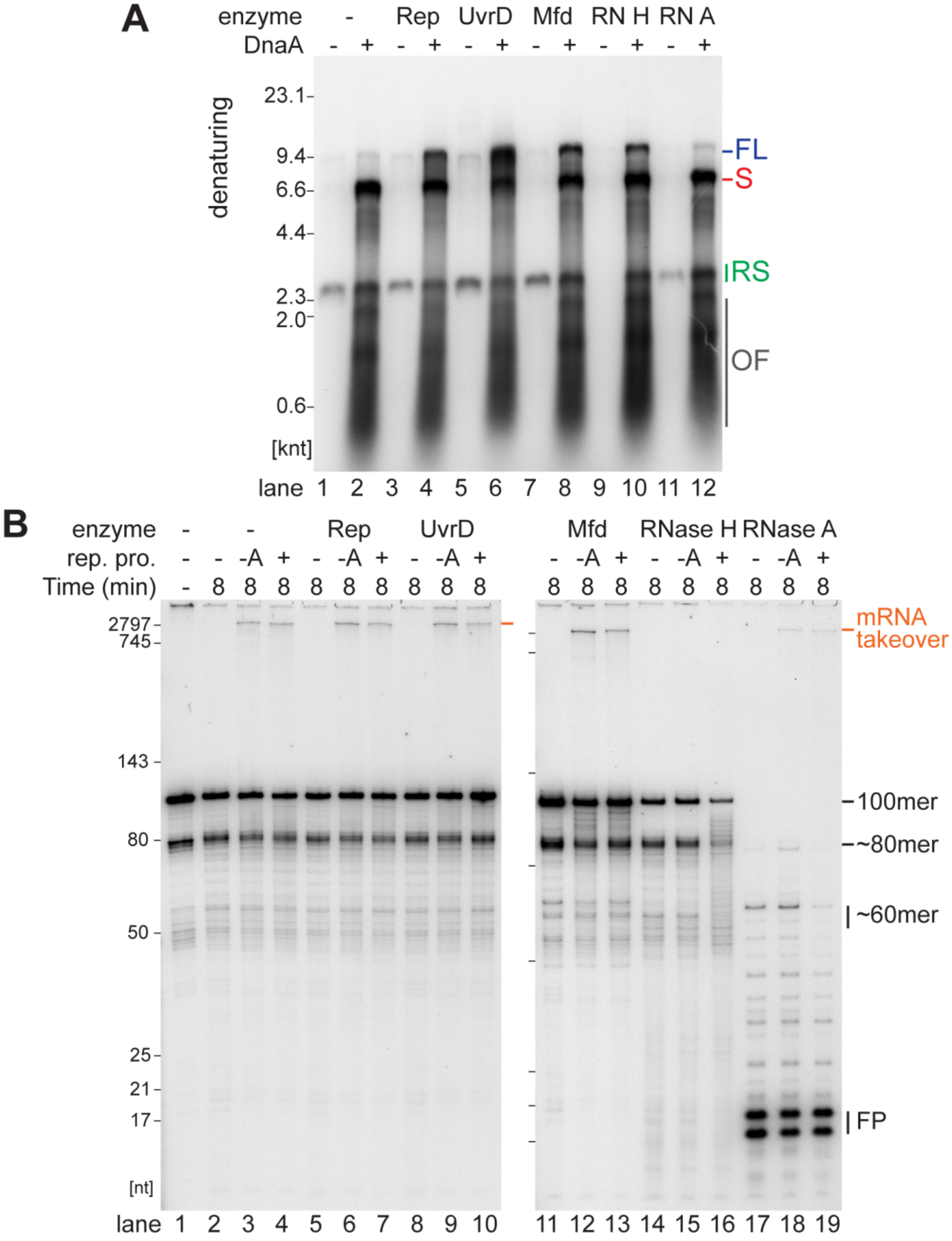

Supplementary Figure 5. Impact of additional factors on replication and mRNA products (related to Fig. 5).

(A) Eight minute replication incubation of the CO DNA-RNAP array (formed on CO_{100}) following a 10 min incubation in the presence of additional factors (same samples and concentrations as in Fig. 5). Reactions either lacked (-) or contained (+) DnaA to visualize *oriC*-independent product formation. Replication products were labeled by the incorporation of [α-^{32}P]dAMP.

(B) The same samples in Figs. 5e and f were analyzed through a 10% 7M urea polyacrylamide gel to monitor the effects of the indicated factors on the [α-^{32}P]GMP-labeled mRNA transcripts. FL, full length; S, leading-strand stall product; RS, leading-strand restart products; rep. pro; replication proteins; FP, RNAP footprint.

Supplementary Figure 6

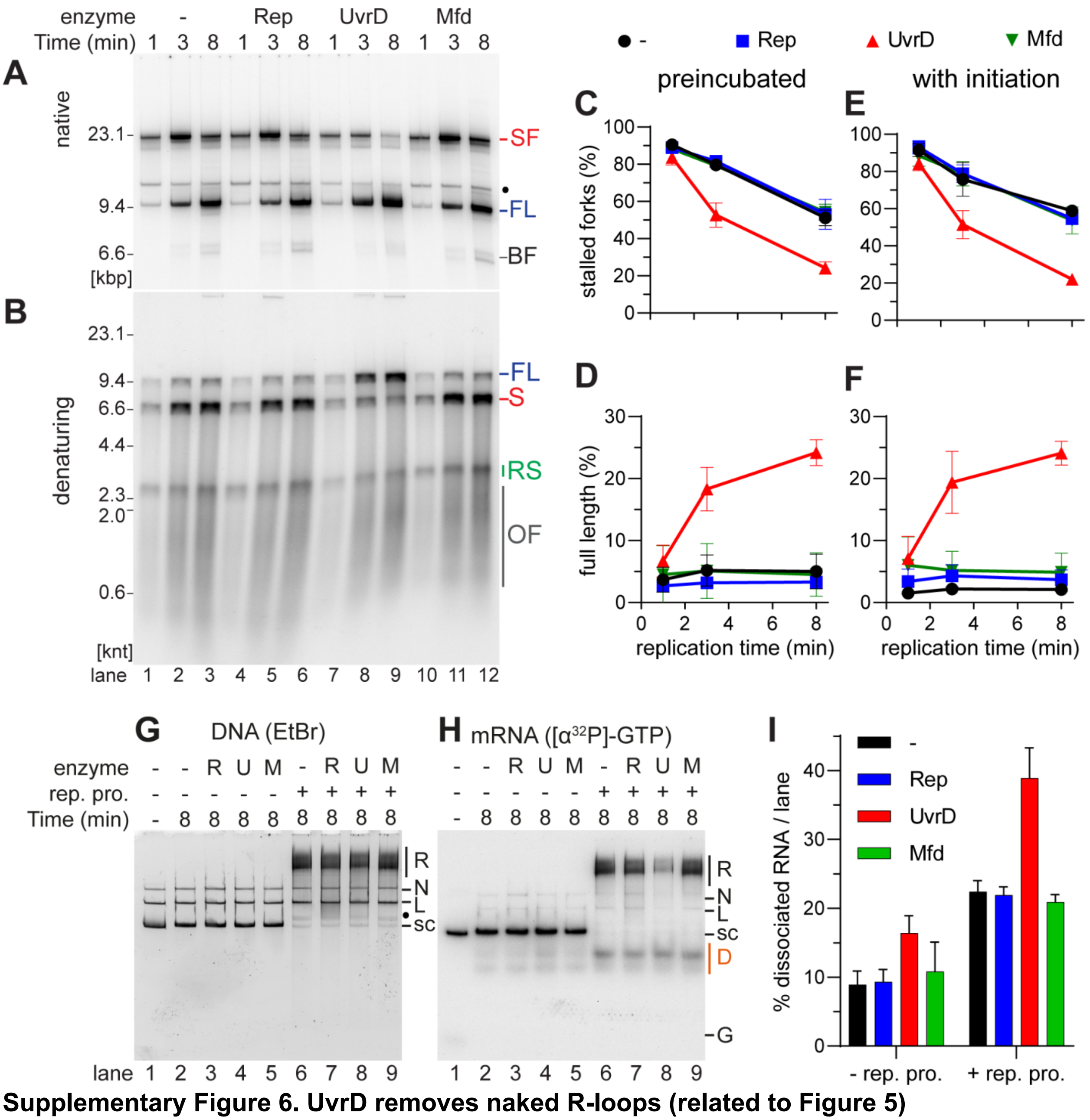

Supplementary Figure 6. UvrD removes naked R-loops (related to Figure 5)
Native (A) and denaturing (B) agarose gels of products in replication time courses of CO replication collisions with R-loop arrays (on template CO_{100}) after the indicated factors were added for 10 min prior to the initiation of replication. Final concentrations were 100 nM Rep (lanes 4-6), 100 nM UvrD (lanes 7-9), 500 nM Mfd (lanes 10-12). Quantification of (C) stalled forks or (D) full-length products (n=3, mean ± standard deviation). (E and F) Quantification of (E)

stalled forks or (F) full length products from the similar experiments as in panels A and B, except that Rep, UvrD and Mfd were not preincubated with the template but were added at replication initiation (n=2, mean ± standard deviation). UvrD displaces RNA from R-loops (G-I) Native agarose gel of replication reaction products **(G)** stained with ethidium bromide or (H) visualized by autoradiography of [α-^{32}P]GMP-labeled mRNA. Pooled, gel filtered DNA-R-loop array complexes (lane 1) were incubated for 10 min with the indicated factors (same concentrations as in panel A). Incubation was continued for another 8 min with the omission of any replication proteins (-) or with the full complement of replication proteins (+). Reactions were terminated by the addition of 30 mM EDTA without any restriction enzyme digestion. Note that no [α-^{32}P]dATP was added to the reactions of panels G and H. (I) Quantification of the fraction of displaced mRNA products in panel H (n=2, mean ± standard deviation).
SF, stalled fork; FL, full length, BF, broken fork; S, leading-strand stall product; RS, leading-strand restart products; OF, Okazaki fragments; R, replicated; N, nicked; L, linear; ●, *oriC*-independent mRNA extension product; s.c., supercoiled; D, displaced mRNA; G, [α-^{32}P]GMP; rep. pro., replication proteins; R, Rep; U, UvrD; M, Mfd.

Supplementary Figure 7

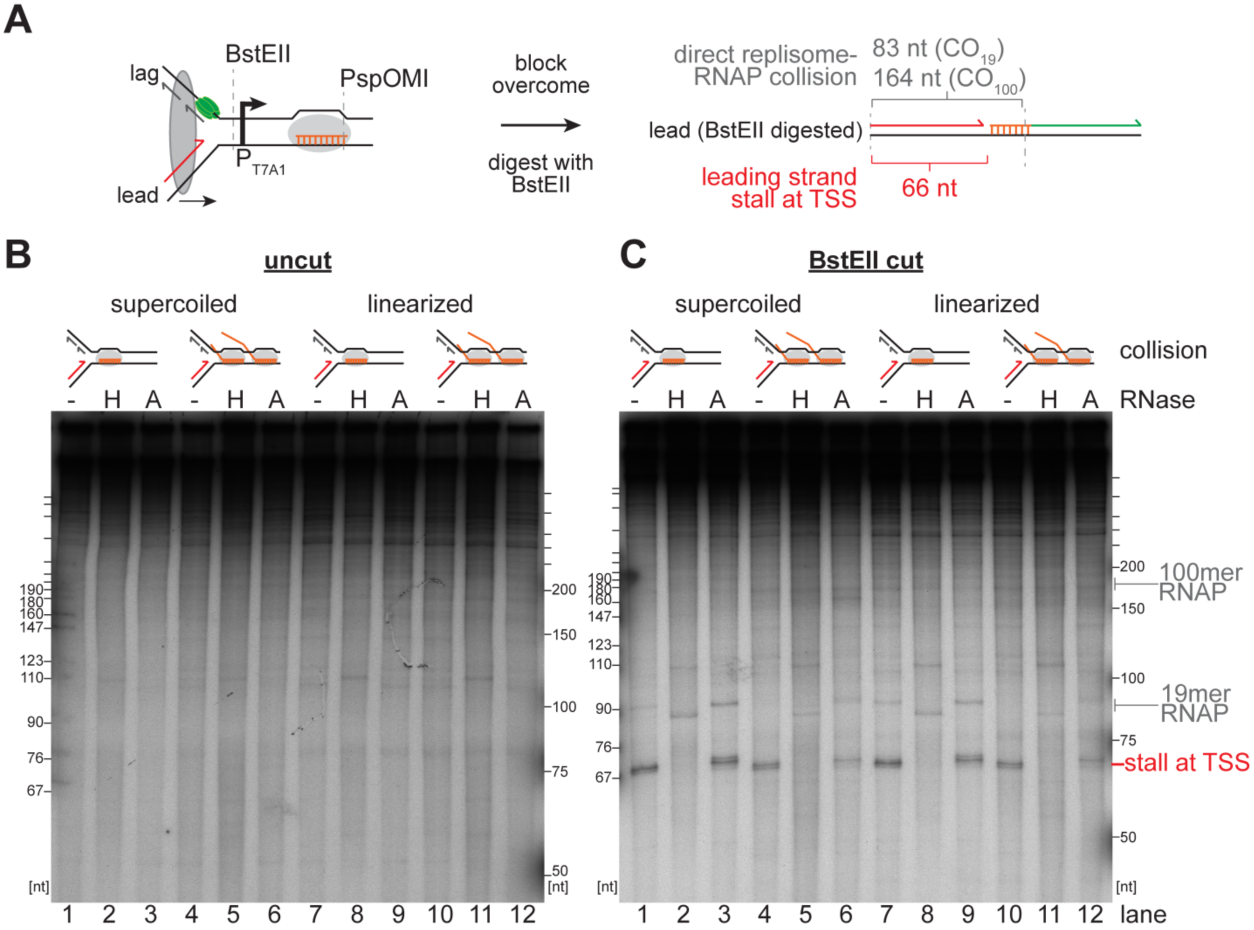

Supplementary Figure 7. Leading-strand synthesis stalls at the transcription start site (related to Fig. 5).
(A) Cartoon indicating the position of relevant restriction enzymes and the lengths of the expected DNA fragments.

Replication reaction products with either a 19mer RNAP (template CO_{19}) or an RNAP array (template CO_{100}) template after 10 min treatment with the indicated RNases were either (B) not digested or (C) were digested with BstEII and analyzed by electrophoresis through a 10% 7M urea polyacrylamide gel. Templates were either left supercoiled for the 8 min reaction time or linearized by ScaI digestion after 1 min (n=3). Size markers are [α-^{32}P]dAMP-labeled pBR322 DNA-MspI Digest (NEB N3032) and Low Molecular Weight DNA Ladder (NEB N3233). Dark grey oval, replisome; light grey ovals, RNAP; TSS, transcription start site.
